# An optimised CRISPR Cas9 and Cas12a mutagenesis toolkit for Barley and Wheat

**DOI:** 10.1186/s13007-024-01234-y

**Published:** 2024-08-13

**Authors:** Tom Lawrenson, Martha Clarke, Rachel Kirby, Macarena Forner, Burkhard Steuernagel, James K. M. Brown, Wendy Harwood

**Affiliations:** John Innes Centre, Norwich Research Park, Norwich, NR4 7UH UK

**Keywords:** Targeted mutagenesis, Genome editing, Cereal, Intron-mediated enhancement, Golden Gate

## Abstract

**Background:**

CRISPR Cas9 and Cas12a are the two most frequently used programmable nucleases reported in plant systems. There is now a wide range of component parts for both which likely have varying degrees of effectiveness and potentially applicability to different species. Our aim was to develop and optimise Cas9 and Cas12a based systems for highly efficient genome editing in the monocotyledons barley and wheat and produce a user-friendly toolbox facilitating simplex and multiplex editing in the cereal community.

**Results:**

We identified a *Zea mays* codon optimised Cas9 with 13 introns in conjunction with arrayed guides driven by U6 and U3 promoters as the best performer in barley where 100% of T0 plants were simultaneously edited in all three target genes. When this system was used in wheat > 90% of T0 plants were edited in all three subgenome targets. For Cas12a, an *Arabidopsis* codon optimised sequence with 8 introns gave the best editing efficiency in barley when combined with a tRNA based multiguide array, resulting in 90% mutant alleles in three simultaneously targeted genes. When we applied this Cas12a system in wheat 86% & 93% of T0 plants were mutated in two genes simultaneously targeted. We show that not all introns contribute equally to enhanced mutagenesis when inserted into a Cas12a coding sequence and that there is rationale for including multiple introns. We also show that the combined effect of two features which boost Cas12a mutagenesis efficiency (D156R mutation and introns) is more than the sum of the features applied separately.

**Conclusion:**

Based on the results of our testing, we describe and provide a GoldenGate modular cloning system for Cas9 and Cas12a use in barley and wheat. Proven Cas nuclease and guide expression cassette options found in the toolkit will facilitate highly efficient simplex and multiplex mutagenesis in both species. We incorporate GRF-GIF transformation boosting cassettes in wheat options to maximise workflow efficiency.

**Supplementary Information:**

The online version contains supplementary material available at 10.1186/s13007-024-01234-y.

## Background

CRISPR (Clustered Regularly Interspaced Short Palindromic Repeats) is now a well-established tool in plant research since the first reports of *Streptococcus pyogenes Cas9* (*Sp*Cas9) use in plants ten years ago [1, 2]. In its simplest form CRISPR is used in targeted mutagenesis, where the position of a double stranded DNA break (DSB) can be precisely controlled, frequently leading to imprecise DNA repair and a resulting loss or change in gene function. Because such mutagenesis is relatively easy to achieve and is amenable to targeting specific loci it has largely replaced random non-targeted methods such as ethyl methanesufonate based techniques. Targeting is programmable via a guide RNA (gRNA) which complexes with the Cas nuclease and contains sequence complementary to the target site.

Researchers have been quick to harness the programmable targeting potential of Cas nucleases to allow a wide variety of other applications including more precise forms of genome editing, where not only the DNA cut site is defined, but also the exact repair outcome. Base and prime editing [3] as well as gene targeting [4] can introduce precise changes ranging in size from a single nucleotide to several kilobases although such edits are often much less frequent events making them harder to achieve. CRISPR nucleases have also been modified to prevent DNA breaks whilst maintaining their ability to bind specific genomic targets, making them useful to target a fused or associated secondary protein to specific loci. Secondary proteins include fluorescent markers to visualise chromosome loci at the sub-nuclear level [5] and enzymes to bring about changes in DNA methylation status, which in some cases have proven to bring about heritable phenotypic changes [6]. Transcriptional activation and repression domains have also been targeted via nuclease dead Cas to bring about upregulation and downregulation of gene expression [7].

**Fig. 1:**
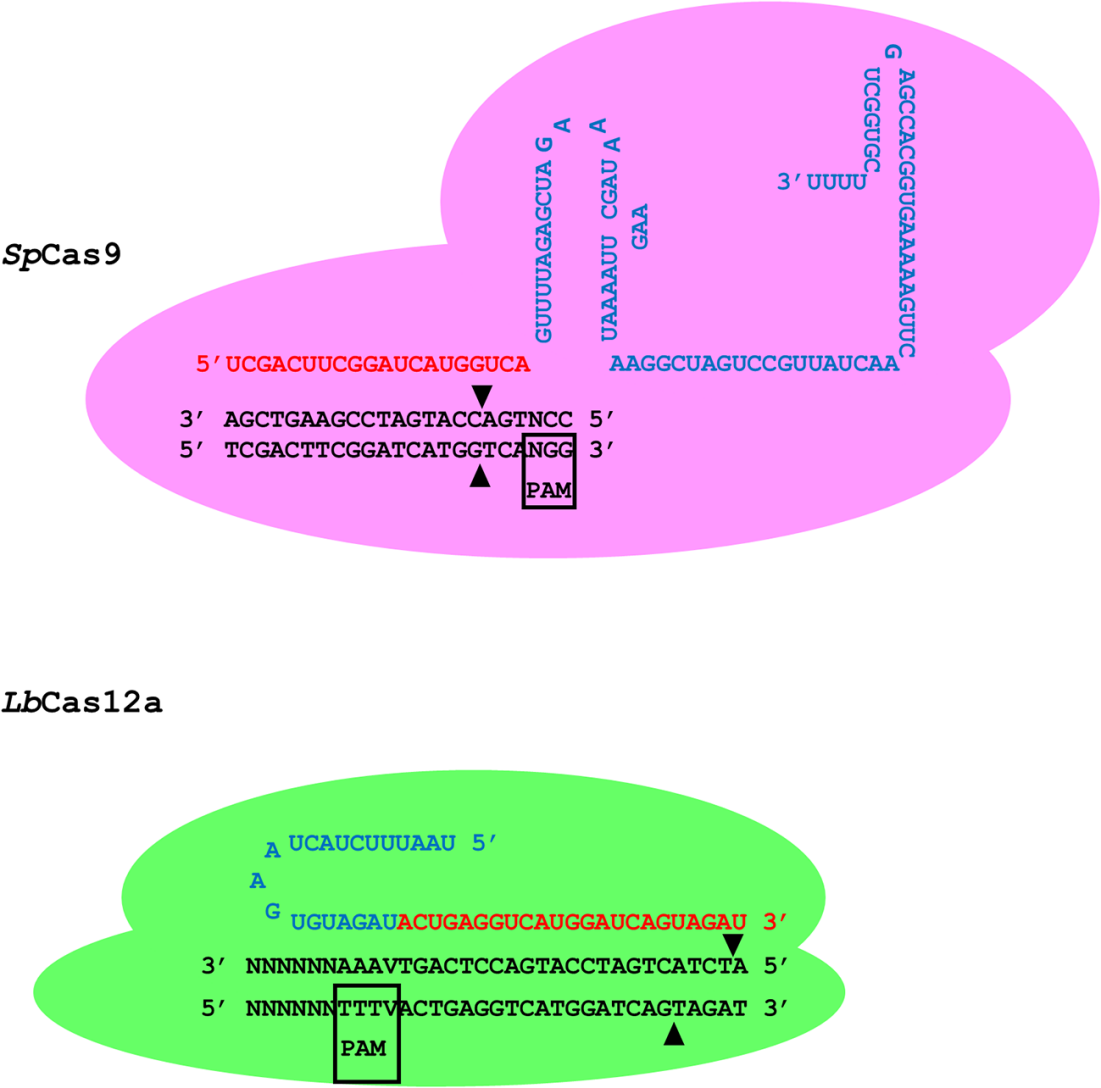
Molecular schematic of Cas9 and Cas12a interaction with genome target. Representation of interaction between Cas9 and Cas12a with target sites in a genome. Black text is a hypothetical target site in double stranded DNA. The PAM is boxed. Red text is the protospacer which is fused to the tracr RNA for Cas9 and a direct repeat (DR) for Cas12a (blue text) in the gRNA. Base pairing between the protospacer and target genomic strand when a PAM is located as shown allows double strand breaks to be made by the Cas nucleases. Position of DNA strand breaks indicated with black triangles

*Sp*Cas9 is the most frequently used Cas nuclease and is now supported by a host of useful tools for selecting gRNA’s [8]. Many of these web-based tools incorporate genome sequences of popular plant species and are particularly useful in avoiding guides which are likely to target undesirable secondary off target loci. Off target sites occur often, particularly in large genomes because of the relatively short sequence giving target specificity in the gRNA. For Cas9 this region of the gRNA, known as the protospacer, is twenty nucleotides long. The protospacer is used to interrogate the genome by Cas9 and upon finding a base pairing match followed by a protospacer adjacent motif (PAM) results in a blunt ended DSB in the genomic target (Fig. 1). However, some mismatches between the protospacer and the genome are tolerated, especially those distal to the PAM and it is here that the guide selection tools are most useful, using data driven algorithms to predict the chance of off target cutting. Unlike off target cutting on target cutting is not reliably predicted by plant guide selection tools currently. For this reason, some researchers pre-validate guides in protoplast or other transient assays. Others, including us, prefer to use multiple guides without pre-validation to insure against low or nonfunctional selections. Of course, when using multiple guides, the potential off-target risk can increase, but this may be addressed by refering to the off targeting predictions created by tools such as the web based CRISPOR.

Cas12a, from *Lachnospiraceae bacterium* (*Lb*Cas12a) is probably the second most widely used CRISPR system in plants after *SpCas9* and has differences in its requirements and outcomes that make it a useful alternative to Cas9 depending on the circumstances [9]. Firstly, the PAM requirement for Cas12a is TTTV which is different to the NGG PAM of Cas9 (Fig. 1). This makes Cas12a particularly useful in GC deserts which are often found in promoter, intron and UTR regions. The Cas12a DSB is staggered compared to the Cas9 blunt cut, which likely explains the larger deletion sizes observed with the former. Recently these features of Cas12a were used to achieve deletion of specific promoter elements in rice [10]. The Cas12a DSB is created at the PAM distal end of the target sequence compared to the PAM proximal cut of Cas9. Because mismatches in the PAM distal end of the target are tolerated more than in the PAM proximal end, it is likely that Cas12a can re-cut imperfectly repaired target DNA. However, one repair error in the Cas9 PAM proximal DSB would not likely be re-cut. Re-cutting of the target site is likely to give further repair outcomes, including those driven by homology directed repair, probably explaining the higher frequency of gene targeting obtained with Cas12a compared to Cas9 [11].

There are a wide variety of *SpCas9* and *LbCas12a* modular parts available, which differ in aspects such as codon usage, presence of introns, promoters and terminators used and the guide RNA expression architecture. This makes the choice of system for new users confusing and prone to sub-optimal results. To maximise efficiency of mutagenesis in wheat and barley, we have recently tested reported component parts and derived modified variants, resulting in highly efficient *SpCas9* and *LbCas12a* systems in these species. We present here the results of our testing leading to the provision of a GoldenGate based toolkit which can be accessed via the AddGene repository facilitating highly efficient Cas9 and Cas12a genome editing in wheat and barley. The toolkit is amenable to producing stable transgenic plants using T-DNA delivery *via Agrobacterium tumefaciens* with reported methods [12, 13]. For wheat we have integrated GRF-GIF factors as an option to maximise transformation efficiency [14].

## Results

### Cas9 cassette optimisation

**Fig. 2:**
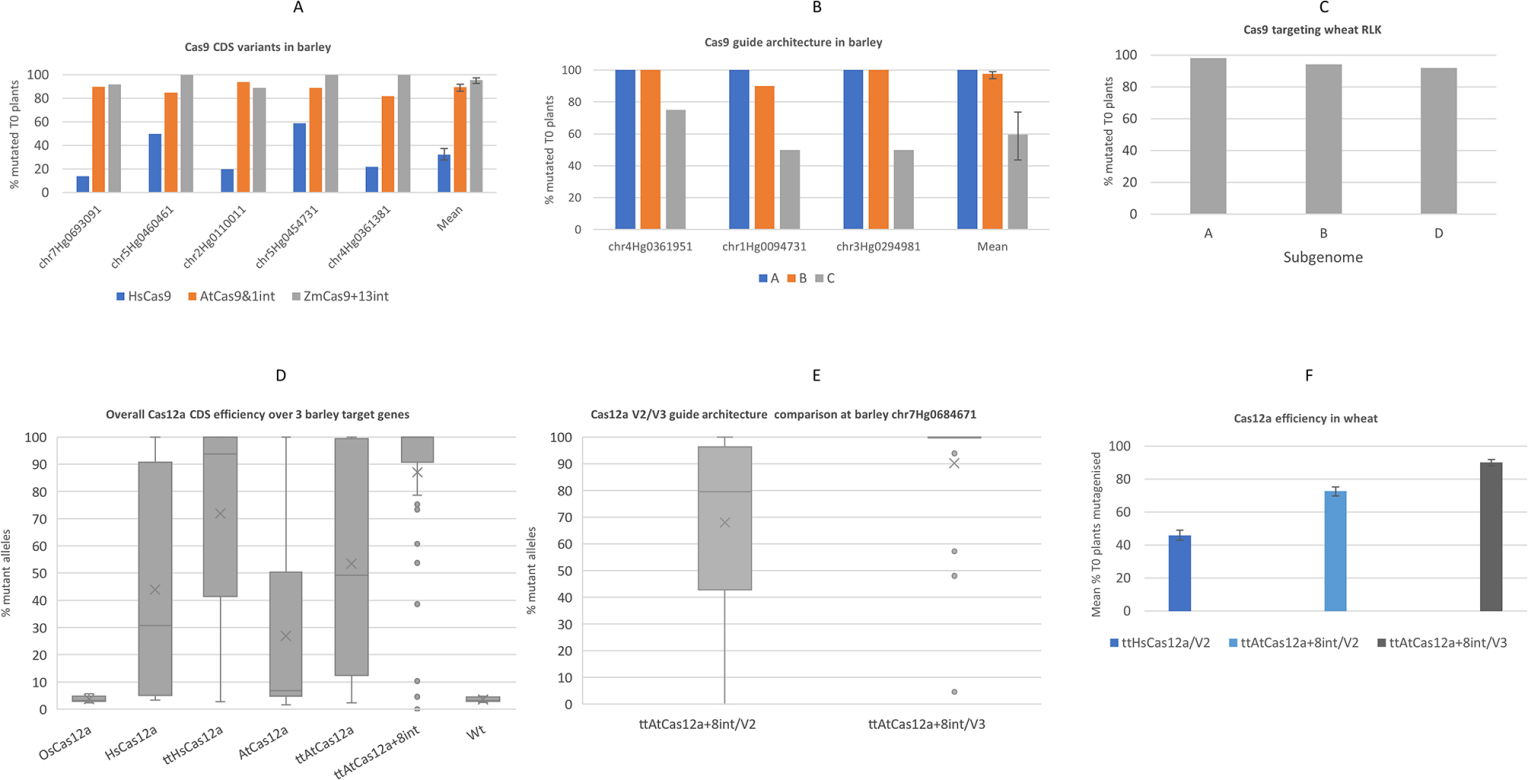
Performance of Cas9 and Cas12a components in barley and wheat. **2A**. Percentage of T0 barley plants containing Sanger sequencing detected mutations in five target genes (chr7Hg0693091, chr5Hg0460461, chr2Hg0110011, chr5Hg0454731, chr4Hg0361381) using three different Cas9 CDS variants. HsCas9 = Human codon optimised CDS, AtCas9 + 1int = Arabidopsis codon optimised Cas9 with 1 intron, ZmCas9 + 13int = Zea mays codon optimised CDS with 13 introns. For the data in Fig. 2A, B, C and F, mutation frequencies were analysed by generalised linear modelling (GLM) of proportions of T0 plants which were mutagenised, using a binomial distribution with a logit link function. In Fig. 2A, in a model of Gene + Cas9 variant, differences between Cas9 variants were highly significant (chi-squared [Chi2] = 65.4, 2 degrees of freedom [df], *P* < 0.001) but variation between genes was not (Chi2 = 1.90, 4 df, *P* = 0.1). Error bars are standard errors of mean efficiency for each construct averaged over genes. **2B**. Percentage of T0 barley plants containing Sanger sequencing detected mutations in three simultaneously targeted genes, using three different Cas9 guide architectures. Architecture A has all 12 guides driven by their own U3 or U6 promoter. Architecture B has a single rice ubiquitin promoter driving all 12 guides which are separated by hammerhead and hepatitis delta virus ribozymes. Architecture C has a single rice ubiquitin promoter driving all 12 guides which are separated by CSY4 cleavage sequences. Csy4 is co-expressed within this architecture. The statistical model was Gene + Architecture; the Architecture effect was very highly significant (Chi2 = 10.9, 2 df, *P* < 0.001) but the Gene effect was not (Chi2 = 1.06, 2 df, *P* = 0.3). Error bars standard errors of mean efficiency for each architecture averaged over genes. **2 C**. Percentage of T0 wheat plants mutagenised in A, B & D subgenomes of target RLK genes using the Cas9 system that worked best in barley (*Zm*Cas9 + 13int/Guide architecture A). Variation in mutation frequencies between genomes was not statistically significant (Chi2 = 1.05, 2 df, *P* = 0.4). **2D**. Overall mutagenesis efficiencies of Cas12a CDS variants over three barley gene targets (chr6Hg0653951, chr7Hg0684671 and chr2Hg0138701). 20 T0 plants were screened for each CDS by PCR and Illumina sequencing at each of the three gene target sites. The percentage of mutant alleles detected for each plant in the three genes are plotted: Median indicated by horizontal bar, mean with an X, boxes contain the first and third quartiles, whiskers extend to a maximum 1.5 times the interquartile range beyond which outliers are marked with a dot. Data on frequencies of mutated alleles in Fig. 2D and E were analysed by analysis of variance (anova). In Fig. 2D, the model used was Gene * CDS (* is the crossing operator) and all three terms were highly significant (Gene: F = 140, 2 numerator df [ndf]; CDS: F = 151, 6 ndf; Gene.CDS: F = 15.7, 12 ndf; 399 denominator df [ddf] and *P* < 0.001 for all three terms). **2E**. Comparison of the mutagenesis efficiencies of Cas12a V2 and V3 guide architectures at the recalcitrant barley target chr7Hg0684671. 20 T0 plants were screened for both V2 & V3 architectures by PCR and Illumina sequencing at the chr7Hg0684671 gene target site. The percentage of mutant alleles detected for each plant are plotted: Median indicated by horizontal bar, mean with an X, box contain the first and third quartiles, whiskers extend to a maximum 1.5 times the interquartile range beyond which outliers are marked with a dot. The statistical model was Gene*CDS and all three terms were highly significant (Gene: F = 78.9, 2 ndf; CDS: F = 90.6, 3 ndf; Gene.CDS: F = 34.6, 6 ndf; 228 ddf and *P* < 0.001 for all three terms). **2 F**. Simultaneous targeting of two wheat genes (*Ta*GW2 & *Ta*GW7) using Cas12a parts proven in barley. The mean percentage of T0 plants mutagenised across the two genes in all subgenomes is shown for the three constructs tested. ttHsCas12a = human codon optimised Cas12a CDS with D156R, ttAtCas12 + 8int = AtCas12a CDS with D156R & 8 introns. V2 = guides in V2 architecture array, V3 = guides in V3 architecture array. Variation between constructs was very highly significant (Chi2 = 68.4, 2 df, *P* < 0.001). Error bars are standard errors of mutagenesis frequencies for each construct averaged over genes and genomes. All statistical analysis was done with the package Genstat 23rd edition (VSN International, U.K.)

Our pilot work in barley [15] utilised a Cas9 coding sequence (CDS) which was codon optimised for expression in human cells (*Hs*Cas9) [16] and whilst functional in barley resulted in rather a low efficiency of mutagenesis (23% of plants for one construct and 10% for a second construct). More recently, other versions of Cas9 CDS’s were reported to work efficiently in other plant species. An *Arabidopsis* codon optimised version with one intron (*At*Cas9 + 1int) [17] outperformed *Hs*Cas9 in *Arabidopsis*. In another report a *Zea mays* codon optimised version with thirteen introns (*Zm*Cas9 + 13int) outperformed *Hs*Cas9 in *Arabidopsis* and was also very effective in *N.benthamiana* and *C. roseus* [11]. To compare these three coding sequence variants in barley, we targeted five genes. Each gene was targeted by four guides, which were paired with either *Hs*Cas9, *At*Cas9 + 1int or *Zm*Cas9 + 13int in three separate constructs. Four guides per gene were used as guide function was not prevalidated. Suitable guides were selected so that off target genes of concern were unlikely to be mutated. In total fifteen constructs were made and transformed into the barley cultivar Golden Promise, enabling CDS comparison over five different target genes (Additional file 1). Between 17 and 24 independent T0 lines were made for each construct, which were subsequently screened by PCR and Sanger amplicon sequencing. Lines were scored positive for mutagenesis where indels or larger fragment insertions/deletions were detected by alignment to wild type reference sequence. Both intron containing versions (*At*Cas9 + 1int & *Zm*Cas9 + 13int) clearly outperformed *Hs*Cas9 (*P* < 0.001), while there was some evidence that *Zm*Cas9 + 13int performed better than *At*Cas9 + 1int (*P* < 0.02) (Fig. 2a & additional file 2). The average mutagenesis efficiency for the three CDS variants over the five genes was 33% (*Hs*Cas9), 88% (*At*Cas9 + 1int) and 96% (*Zm*Cas9 + 13int).

### Cas9 guide architecture optimisation

Most frequently guide RNAs for Cas9 are expressed using a polymerase 3 promoter, typically with one promoter per guide. Polymerase 2 promoters can be used to successfully drive guide expression in plants, where a single promoter can drive a multiple guide array. In this situation all guides are transcribed on a single transcript and are separated by sequences which bring about precise cleavage of the transcript to release individual guide RNAs, enabling Cas9/guide ribonucleoprotein formation. One way to release guides from a single transcript has been to use self-cleaving ribozyme sequences, which has been shown in Cas12a systems [18]. Another way to bring about transcript processing is to separate each guide with the recognition/cleavage sequence for CSY4, a CRISPR associated protein, which is co-expressed with the Cas nuclease and is therefore able to release individual guides. This system is reported to work very effectively in some dicotyledonous species [19–21]. We decided to compare these three different guide architectures for expressing Cas9 guides in barley, this time by targeting three genes simultaneously. Three constructs were made, differing only in the guide architecture used (Additional file 3). The protospacers used in each architecture were identical and in the same sequential order for the three constructs. Each of the three genes was targeted by 4 different gene specific guides, meaning each construct contained 12 guides. In the first construct (A), each of the 12 guides was driven by its own polymerase 3 promoter, either *Ta*U6, *Ta*U3 or *Hv*U3. In the second construct (B) the 12 guides were driven by a single *Os*Ubiquitin polymerase 2 promoter and separated by self-cleaving hammerhead (HH) and hepatitis delta virus (HDV) ribozyme sequences. In the third construct (C) the 12 guides were again driven by a single *Os*Ubiquitin polymerase 2 promoter but this time separated by CSY4 recognition sequences. The CSY4 protein was also co-expressed in this construct to bring about transcript processing. The Cas9 variant used in all 3 constructs was *Zm*Cas9 + 13int. We produced 20 T0 lines for constructs A and B, but despite multiple repeat transformations, were only able to produce 4 T0 lines for construct C. Architecture A was the most efficient where 100% of T0 plants were mutagenised in all three of the target genes (Fig. 2b & additional file 4). Architecture B was almost as efficient where 100% of T0 plants were mutagenised in two of the target genes and 90% were mutagenised in the third gene. Architecture C was the least efficient, with 75% of T0 plants mutagenised in one of the target genes and 50% mutagenised in two target genes. The mean mutagenesis efficiencies of Cas9 guide architectures A, B and C were 100%, 97% and 58% respectively. Generalised linear modelling showed that architectures A and B were significantly more mutagenic than C (*P* < 0.001), but there was only a slight difference between A and B (*P* = 0.09).

### Cas9 in wheat

We took the best performing Cas9 parts from the barley experiments above (*Zm*Cas9 + 13int & guide architecture A) and used them to target a single receptor like kinase gene (*RLK*) in hexaploid wheat (*cv*. Fielder) using a single construct (Additional file 5). Four guides each targeting the A, B & D copies (TraesCS7A02G264400, TraesCS7B02G162500, TraesCS7D02G265400) were used. In addition, a GRF-GIF overexpression cassette was included [14] to boost transformation efficiency. Forty-eight transgenic lines were screened by PCR/Sanger sequencing at each of the A, B & D target sites in conjunction with alignment to wild type reference sequences to identify mutagenic lines. The mutagenesis efficiency was 98%, 94% & 92% in the A, B & D subgenomes respectively with an overall mean of 95% (Fig. 2c & additional file 6).

### Cas12a CDS optimisation

The first report of *LbCas12a* use in plants was in rice where the CDS used (*Os*Cas12a) was codon optimised for that species [22]. Later a CDS which was originally used in human cells and codon optimised accordingly (*Hs*Cas12a) [23] was shown to work in *N.benthamiana*, tomato and *Arabidopsis* [24]. A third version was codon optimised for *Arabidopsis* and was shown to be considerably more efficient for mutagenesis at the relatively low temperatures plants grow at (tt = temperature tolerant), when a D156R mutation was included (tt*At*Cas12a) [25]. We decided to compare these CDS variants in barley in addition to three further versions, firstly tt*At*Cas12a without the temperature tolerant mutation (*At*Cas12a), secondly *Hs*Cas12a containing the D156R mutation (tt*Hs*Cas12a), and thirdly tt*At*Cas12a into which we inserted eight *Arabidopsis* introns (tt*At*Cas12a + 8int). To compare these 6 Cas12a CDS’s, we made six constructs (Additional file 7) differing only in the CDS used. Each construct contained a 3 guide array targeting the barley genes chr6Hg0653951, chr7Hg0684671 and chr2Hg0138701 using a guide architecture shown to function in *Arabidopsis* [26] The protospacers were prevalidated in transgenic plants by us and so just one guide was used for each target gene. Twenty T0 plants were made for each of the six constructs which were screened at the target loci by Illumina amplicon sequencing. The percentage of mutant reads was determined at each of the three target loci for each T0 plant produced (Additional file 8). Figure 2d shows the overall mutagenesis efficiencies of the Cas12a CDS variants over the three target genes. Surprisingly the *Os*Cas12a was not functional in barley with no increase in the mutations detected compared to the wild type control (mean overall background artifact mutations of 3.6% and 3.5% respectively). *At*Cas12a had a mean efficiency of 26% although chr7Hg0684671 was not hit much above the baseline of the WT control (*At*Cas12a = 5%, WT = 4.5%) (Additional files 8 & 9). *Hs*Cas12a was more effective than *At*Cas12a overall (*P* < 0.001), having a mean efficiency of 44% although still it was ineffective at targeting chr7Hg0684671. Addition of the D156R temperature tolerant mutation in both *Arabidopsis* and Human optimised CDSs significantly increased the overall mean mutagenesis efficiency to 53% and 72% for tt*At*Cas12a and tt*Hs*Cas12a respectively (*P* < 0.001 in both cases). The D156R addition also enabled the mutagenesis of chr7Hg0684671 with tt*At*Cas12a and tt*Hs*Cas12a to 17% and 33% respectively. Overall tt*At*Cas12a + 8int performed best with a mean overall efficiency of 87%, which was significantly better than tt*At*Cas12a (*P* < 0.001), the same CDS without introns and tt*Hs*Cas12a (*P* < 0.001), the next best performing CDS. Even the difficult to target chr7Hg0684671 was mutagenised at a mean efficiency of 68%.

### Dissecting the role of introns in Cas12a

We decided to dissect the role that the eight introns inserted into tt*At*Cas12a + 8int played in boosting the performance of tt*At*Cas12a. To do this we made a further four constructs which retained some of the introns contained in tt*At*Cas12a + 8int. tt*At*Cas12a + 1int retained just the first intron, tt*At*Cas12a + S1int the first 3 introns, tt*At*Cas12a + S2int the second 3 introns and tt*At*Cas12a + S3int the last 2 introns (Additional file 10). These four additional Cas12a variants were coupled with the guide cassettes targeting the same three barley genes as before (chr6Hg0653951, chr7Hg0684671 and chr2Hg0138701) and once again twenty T0 lines were produced and screened using Illumina amplicon sequencing. Inclusion of the first intron in tt*At*Cas12a + 1int had no great boosting effect with a similar overall mean efficiency to the no intron version tt*At*Cas12a of 54% & 53% respectively (*P* = 0.9) (Additional files 10 & 11). The first three introns in tt*At*Cas12a + S1int enhanced overall efficiency by 14% (*P* = 0.006), the second three introns in tt*At*Cas12a + S2int by 31% (*P* < 0.001) and the final 2 introns in tt*At*Cas12a + S3int by 13% (*P* = 0.007). The majority of intron mediated enhanced mutagenesis was because of the 3 introns in tt*At*Cas12a + S2int which has a very similar overall percentage enhancement compared to the 8 introns in tt*At*Cas12a + 8int across all three genes (31% & 34% respectively, *P* = 0.6). However, comparing the percentage enhancement of tt*At*Cas12a + 8int and tt*At*Cas12a + S2int at the most recalcitrant target chr7Hg0684671 (51% and 43% respectively), shows there is a greater but not statistically significant additional enhancement of 8% with all eight introns compared to just the three in tt*At*Cas12a + S2int (*P* = 0.3) (Additional files 10 & 11).

### Combinatorial effect of D156R and introns in Cas12a

We also wanted to see if there was any combined advantage with the inclusion of introns and the D156R mutation which was greater than the sum of the individual parts. To do this we required one further construct targeting the three barley genes chr6Hg0653951, chr7Hg0684671 and chr2Hg0138701. This construct had a wild type D156 residue and all eight introns, being designated *At*Cas12a + 8int. By comparing the efficiency of *At*Cas12a to tt*At*Cas12a, *At*Cas12a + 8int and tt*At*Cas12a + 8int the separate and combined enhancing roles of D156R and introns could be determined. Overall mean efficiencies (Additional files 12 & 13) were improved by the inclusion of singular D156R (53%) and introns (55%) in tt*At*Cas12a & *A*tCas12a + 8int relative to *At*Cas12a (26%), the CDS which contained neither. The overall mean efficiency when both D156R and introns were combined in tt*At*Cas12a + 8int was highest at 87%. For the target genes chr6Hg0653951 and chr2Hg0138701 the mean efficiencies of singular DI56R (tt*At*Cas12a) and introns (*At*Cas12 + 8int) were too high to allow determination of the sum of the individual parts (D156R enhancement + intron enhancement + base *At*Cas12a efficiency) because these values were more than 100% (Additional file 13). However, the sum of the enhancements in efficiency for the recalcitrant target chr7Hg0684671 gained by D156R and the introns separately was just 20%, allowing the synergistic gain from the combined effect of D156R and the introns to be determined as 48% (*P* < 0.001), considerably higher than the sum of the separate enhancements (Additional file 12 and 13).

### Cas12a guide architecture optimisation

We already achieved efficient Cas12a mutagenesis in the CDS comparison above using a ribozyme-based guide architecture we designate as version 2 (V2). Previously we tested a version 1 (V1) architecture which relies on the innate processing activity of Cas12a itself to release individual guides from a single transcript and found V2 superior to V1 [27]. Later, it was reported that a Cas12a guide architecture containing tRNA sequence instead of ribozymes worked best in wheat [28] and so we decided to test this in barley, designating the tRNA version V3. For this we used two constructs each targeting the same three genes that were used in the Cas12a CDS comparison (chr6Hg0653951, chr7Hg0684671 and chr2Hg0138701). One construct contained guides in a V2 array and the other a V3 array (Additional file 14). Twenty T0 plants were made for each construct which were screened by Illumina amplicon sequencing to determine the percentage mutation at each locus. Overall, there was little difference in the mean editing efficiency between V2 and V3 over the three targets which recorded 87% and 90% respectively (*P* = 0.5; Additional file 15). However, the mean efficiencies were significantly different (*P* = 0.009) at the recalcitrant chr7Hg0684671 locus with V2 and V3 values of 68% and 90% respectively (Fig. 2e), showing a 22% improvement when using V3. Differences in the efficiency of V2 and V3 at chr6Hg0653951 & chr2Hg0138701 were not statistically significant (*P* = 0.9 and *P* = 0.1 respectively).

### Cas12a in wheat

We trialled two Cas12a CDS’s which performed well in barley (tt*Hs*Cas12a & tt*At*Cas12a + 8int) in wheat. To do this we selected three guides which were previously shown to be effective at targeting two genes in hexaploid wheat, GW2 and GW7 [29]. Guides GW7T14 and GW7T13 target the three subgenome copies of GW7 and GW2T6 targets the three subgenome copies of GW2. Two constructs were made differing only in the Cas12a CDS used which contained these three guides in a V2 guide architecture (Additional file 16). A third construct was also made in response to the recent report [28] which identified a tRNA based guide architecture (V3) as being superior to a V2 architecture in wheat. Additionally, all three constructs contained a GRF-GIF overexpression cassette to increase the transformation efficiency [14]. Constructs were transformed into the spring variety Fielder and 48 lines per construct were screened at the target loci by Sanger amplicon sequencing and alignment to the wild type reference. As was seen in barley, tt*At*Cas12a + 8int performed better than tt*Hs*Cas12a (Fig. 2f & additional file 17) in wheat, giving at all subgenome GW2 and GW7 loci a greater percentage of mutagenised T0 plants. The average percentage of mutagenised T0 plants across all subgenomes of both GW2 and GW7 was 46% for tt*Hs*Cas12a and 73% for tt*At*Cas12a + 8int with the V2 guide architecture (*P* < 0.001). Comparison of the mutagenesis efficiency resulting from the two guide architectures shows that with the tt*At*Cas12a + 8int CDS, V3 outperforms V2 in 5 of the 6 subgenome loci and raises the average percentage of mutagenised T0 plants from 73 to 90% (*P* < 0.001).

### Cas9 and Cas12a co-mutagenesis in Barley

We tested whether it was possible to introduce both Cas9 and Cas12a components on the same T-DNA to edit simultaneously in barley. To do this, we made constructs expressing both *Zm*Cas9 + 13int and tt*At*Cas12a + 8int in addition to guide expression cassettes of type A for Cas9 and V2 for Cas12a (Additional file 18). Four constructs were made, two targeting two genes (constructs 1 & 2), one targeting three genes (construct 3) and one targeting four genes (construct 4). Each gene was targeted by four different gene specific guides, either four Cas9 guides, four Cas12a guides, or a combination of Cas9 and Cas12a guides (Table 1 Cas9 and/or Cas12a guides). This made the T-DNA inserts rather large (construct 4 targeting 4 genes had a T-DNA of 28.1 Kb) which likely impacted transformation efficiency. However, we still managed to produce between 11 and 19 T0 plants for each construct, which were screened by Sanger amplicon sequencing and alignment to the wildtype reference. Table 1 shows that with all four constructs Cas9 and Cas12a were able to effectively mutagenise all target genes simultaneously. In T0 lines where Cas9 was effectively editing, it was apparent that Cas12a was also editing. For example, with construct 1, target chr5Hg0454731 addressed by Cas12a was edited in 45% of T0 lines created. Target chr4Hg0361381 addressed by Cas9 in construct 1 was also effective in exactly the same T0 lines, giving the same 45% efficiency at this target. This explains the overall 45% efficiency at “all targets” i.e. the percentage of T0 plants where both targets were simultaneously edited. This pattern was repeated with the remaining constructs 2, 3 and 4 and although not every target gene was hit in each active T0 line, the percentage edited in all targets was always determined by the target with the lowest editing efficiency for that construct. For example, construct 4 targeted chr1Hg0000251 at 68%, chr5Hg0508401 at 90%, chrHg0829711 at 95% and chr3Hg0346691 at 90%. The lowest efficiency for these four targets was 68% for chr1Hg0000251, limiting the percentage of T0 lines where all four genes were successfully targeted to 68%.

**Table 1:**
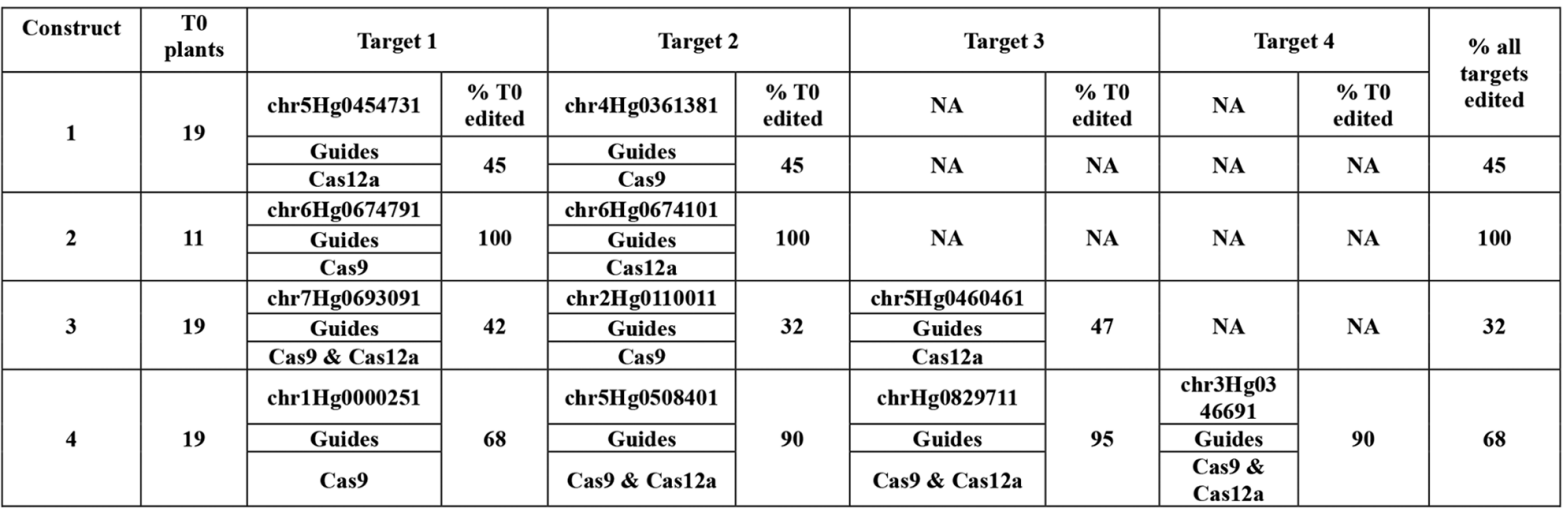
Barley T0 editing efficiency of constructs 1,2,3 & 4 which co-express Cas9 and Cas12a components

### The toolkit

**Fig. 3:**
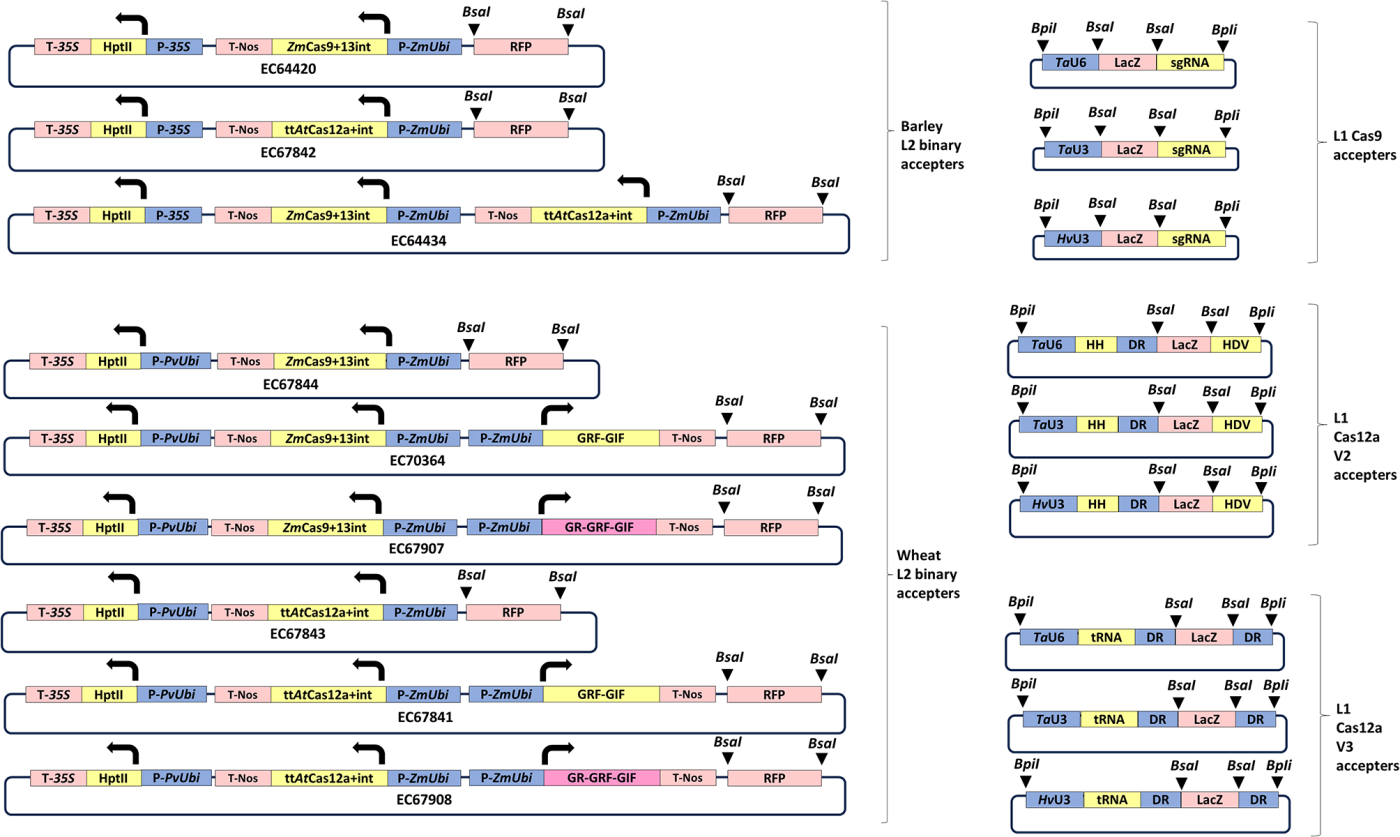
Cas9 and Cas12a toolkit parts available via AddGene. Plasmid parts of the toolkit: Level 1 guide accepters for Cas9 and Cas12a with three promoter options. Compatible binary vectors containing plant selection markers and nuclease cassettes which are wheat or barley specific. P-*35S* = Cauliflower mosaic virus long 35S promoter, P-*Pv*Ubi = *Panicum virgatum* ubiquitin promoter, HptII = hygromycin phosphotransferase CDS with intron, T-*35S* = Cauliflower mosaic virus terminator, P-*Zm*Ubi = *Zea mays* ubiquitin promoter, tt*At*Cas12a + 8int = temperature tolerant *Arabidopsis* codon optimised Cas12a CDS with 8 introns, *Zm*Cas9 + 13int = *Zea mays* codon optimised Cas9 with thirteen introns, T-Nos = nopaline synthase terminator, GRF-GIF = Growth-regulating factor 4 (GRF4) and its cofactor GRF interacting factor 1(GRF1) fusion, GR-GRF-GIF = Rat glucocorticoid receptor (GR) & Growth-regulating factor 4 (GRF4) & its cofactor GRF interacting factor 1(GRF1) fusion. RFP = red fluorescent protein for colour selection during cloning, *Ta*U6 = *Triticum aestivum* U6 promoter, *Ta*U3 = *Triticum aestivum* U3 promoter, *Hv*U3 = *Hordeum vulgare* U3 promoter, HH = Hammerhead ribozyme, HDV = hepatitis delta virus ribozyme, tRNA = tRNA sequence, DR = short invariable 5’ region of Cas12a guide, sgRNA = invariable 3’ region of Cas9 guide, LacZ = blue/white colour selection marker for cloning, *BsaI* = restriction site, *BpiI* = restriction site. Triangles indicate GoldenGate cloning site cut points. Arrows indicate direction of transcription

We tested various Cas9 and Cas12a CDS variants and guide architecture designs in barley then applied the best to wheat, achieving very efficient mutagenesis. These components are the basis for a complete CRISPR Cas9 & Cas12a toolkit we have made available via AddGene. Species specific level 2 binary vectors are provided in the toolkit (Table 2) as well as level 1 guide accepters (Fig. 3).

**Table 2:** Summarises the level 2 binary vectors available

Requirement	Vector								
	EC64420	EC67842	EC64434	EC67844	EC70364	EC67907	EC67843	EC67841	EC67908
Barley	✓	✓	✓	×	×	×	×	×	×
Wheat	×	×	×	✓	✓	✓	✓	✓	✓
Cas9	✓	×	✓	✓	✓	✓	×	×	×
Cas12a	×	✓	✓	×	×	×	✓	✓	✓
GRF-GIF	×	×	×	×	✓	×	×	✓	×
GR-GRF-GIF	×	×	×	×	×	✓	×	×	✓

For barley we provide three level 2 binary vectors containing a hygromycin marker for plant selection and either one or two nuclease expression cassettes. EC64420 contains a single *Zm*Cas9 + 13int expression cassette, EC67842 contains a single tt*At*Cas12a + 8int expression cassette and EC64434 contains both *Zm*Cas9 + 13int & tt*At*Cas12a + 8int expression cassettes. For wheat we provide six level 2 binary vectors containing a hygromycin marker for plant selection and either a *Zm*Cas9 + 13int (EC67844, EC70364, EC67907), or a tt*At*Cas12a + 8int (EC67843, EC67841, EC67908) expression cassette. Each of the nuclease versions is also available with (EC70364 & EC67841) and without (EC67844 & EC67843) an additional GRF-GIF overexpression cassette which greatly increases the efficiency of transformation in many wheat cultivars, but also is associated with some pleiotropic effects including sterility in some varieties. To retain the benefits of GRF-GIF overexpression during the transformation process, whilst alleviating pleiotropic effects later in plant development, we developed two further Cas9 and Cas12a versions possessing GR-GRF-GIF fusions (EC67907, EC67908). These allow induction of GRF-GIF activity in the presence of dexamethasone (Dex), which can be provided in the tissue culture media. Later absence of Dex during growth on soil should eliminate or reduce any undesirable GRF-GIF phenotypes. To derive the GR-GRF-GIF fusion we tested both N-terminal and C-terminal fusions of the rat glucocorticoid receptor (GR) to GRF-GIF (GR-GRF-GIF, GRF-GIF-GR) in two Fielder transformation experiments (Additional file 19). We found that the N-terminal version showed a greater benefit to the number of wheat shoots regenerated in the presence of Dex than the C-terminal version and a greater ability to switch off in the absence of Dex. GR-GRF-GIF gave an average transformation efficiency over two experiments of 55% in the presence of Dex and 15% in the absence of Dex. GRF-GIF-GR gave an average transformation efficiency of 33% with Dex and 24% without Dex. Therefore, the N-terminal fusion gave a 22% higher transformation rate in the presence of the inducer than the C-terminal version (55%>33%) and a greater difference in transformation efficiency between the induced and non-induced states (40%>9%). Whilst it is important to have a high rate of transformation in the presence of the inducer, it is also important that the non-induced condition has a considerably lower rate of transformation, indicating that the GRF-GIF effect is indeed switchable and likely to reduce pleiotropic effects later in development where the inducer is not present. We grew ten of the regenerated Fielder wheat plants containing GR-GRF-GIF taken from the + Dex conditions to maturity and observed no obvious phenotypic effects. All ten had normal spike development and were completely fertile. From preliminary tests in barley, we have not observed any benefit to regeneration of transgenic shoots by overexpressing GRF-GIF from the incoming T-DNA despite an apparent enhancement of callus proliferation. For this reason, the barley binary vectors described above do not contain GRF-GIF options.

**Fig. 4:**
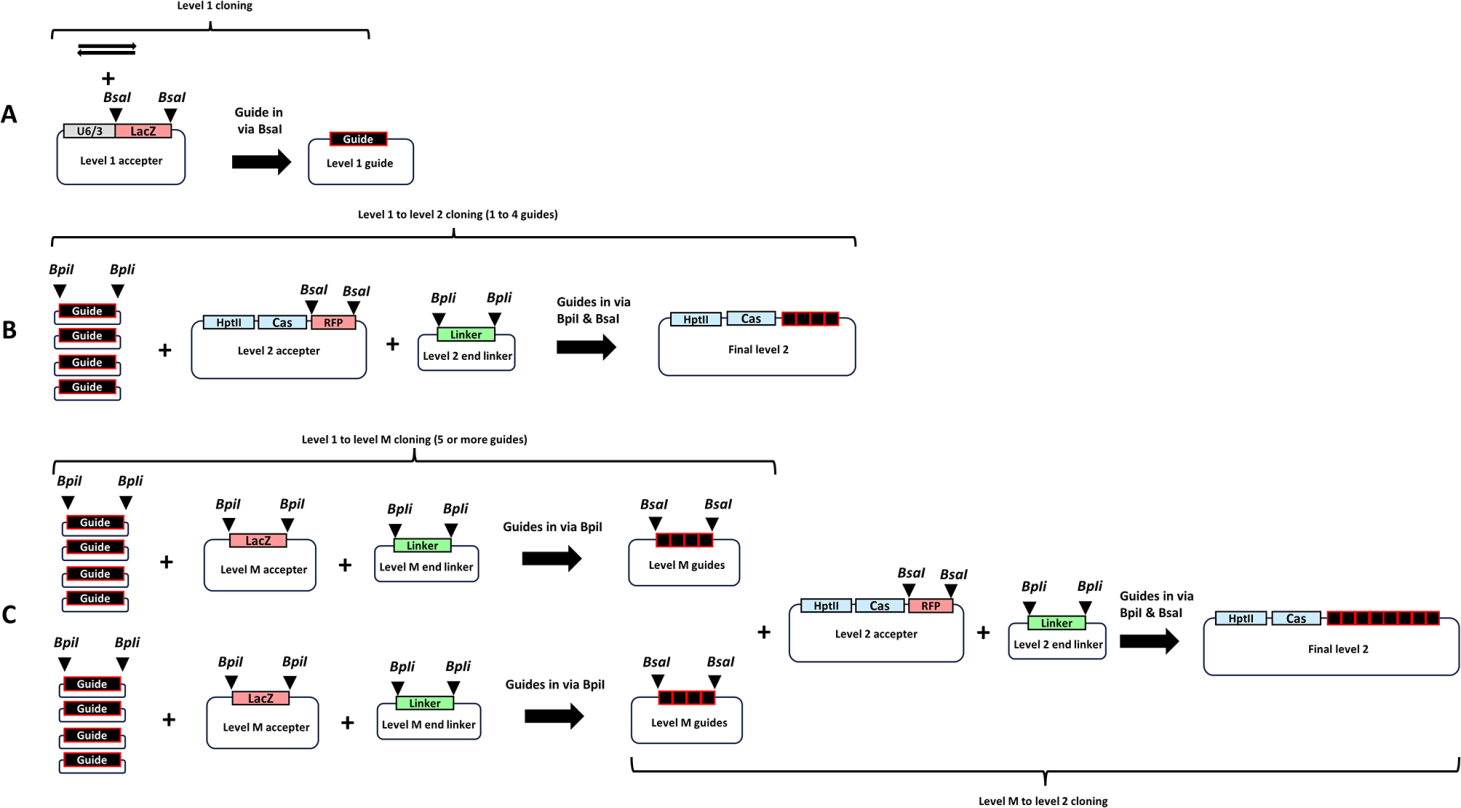
Toolkit cloning strategy. Toolkit main cloning routes: A = level 1 oligo cloning to give guide cassettes (black boxes with red outline), B = Up to 4 level 1 guides directly cloned into level 2 accepter, C = more than 4 guides cloned into level 2 accepter via intermediate level M step. Thin arrows represent complementary oligonucleotides that will later transcribe as the protospacer. Thick arrows show the direction of cloning. U6/3 = U6 or U3 promoter (Note – the invariable DR of Cas12a guide and sgRNA of Cas9 guide are not shown in level 1 guide accepters, but they are present outside of the *BsaI-BsaI* fragment. LacZ = blue/white colour selection marker for cloning, RFP = red fluorescent protein for colour selection during cloning, *BsaI* = restriction site, *BpiI* = restriction site. Triangles indicate GoldenGate cloning site cut points, HptII = hygromycin phosphotransferase plant selection marker, Cas = Cas9 or Cas12a nuclease cassette

For the toolkit, cloning begins at level 1 (Fig. 4a) by the insertion of complementary oligonucleotide pairs representing the protospacer sequence into level 1 guide accepters. Once oligonucleotides have been inserted into the guide accepters the entire transcriptional units can then be cloned either directly into level 2 binary vectors (Fig. 4b), or into level M accepters as an intermediate step (Fig. 4c). We have found that cloning is most efficient when the number of fragments ligated in any one step are kept to a minimum. When between 1 and 4 guides are required, level 1 can proceed directly to level 2 with no intermediate step. When more than 4 guides are required in the final level 2 vector, then the intermediate level M step is used, with again up to 4 guides stacked in any level M vector. Level 1 guide accepters with *Ta*U6, *Ta*U3 and *Hv*U3 promoters are provided in all seven Golden Gate positions, both for the Cas9 guide architecture A (Additional file 3) and Cas12a V2 architecture (Additional file 14). In addition, we also provide Cas12a V3 guide accepters (Additional file 14) in all three promoter & seven position formats. We have found that when stacking level 1 guide cassettes in level M and level 2 it is good practice to alternate the sequential order of U3 and U6 promoters to avoid issues with bacterial recombination which are caused during cloning by repetitive tandem arrays.

Additional file 20 details (in the appropriate tabs) the various level 1, level M, level 2 & end linker plasmids available via AddGene. Genbank (.gb) file downloads of these plasmids are available on the AddGene browser (view these and not the main page plasmid map which does not show the important annotations). Additional file 20 also indicates the appropriate vector choices when between 1 and 26 guides are required in the final level 2 assembly (vector choices tab).

Additional file 21 details the constructs, guides and PCR primers used in this study.

Additional files 22–61 are the gb files of the constructs listed in additional file 21.

## Methods

### Cloning

1. Novel cloning parts were designed in accordance with MoClo [30] compatibility and commercially synthesised. Level M accepters, end linkers and level 2 end linkers were already available via AddGene.
2. Eight short *Arabidopsis* introns were inserted into the tt*At*Cas12a sequence to derive the tt*At*Cas12a + 8int CDS. This was done using the Netgene2 splicing tool in accordance with a previous report [11].
3. Golden Gate cloning reactions contained 100ng of accepter vector with an appropriate mass of all other modules in a reaction to give a molar ratio of 3:1 insert: vector. 10 units of *BsaI* (NEB) and *BpiI* (Thermo Fisher) restriction enzymes were used where appropriate, in conjunction with 400 units of T4 ligase (NEB). Reactions also contained 1x T4 ligase buffer, 0.1 mg/ml BSA and were conducted in a final volume of 15 µl. Reactions were thermocycled twenty-six times for 3 min at 37 °C/3 minutes at 16 °C. Enzymes were then killed using additional steps of 50 °C for 3 min followed by 80 °C for 3 min. Half of each reaction was immediately transformed into Library efficient competent *E.coli* DH5a cells (Thermo Fisher) according to manufacturer instructions. Cells were plated onto appropriate antibiotic containing Luria broth-agar (LBA) plates: Carbenicillin 100 mg/L for level 1, Spectinomycin 100 mg//L for level M and Kanamycin 50 mg/L for level 2. For level 1 and level M, X-Gal to a final concentration of 20ug/ml was added to the molten LBA prior to pouring to aid in selection of white and not blue colonies for screening. This is unnecessary in level 2, where useful white colonies are distinguished from the original accepter vector which contains an RFP cassette giving red colonies. Table 3 shows which restriction enzymes were used at each cloning step relevant to the toolkit.
4. For level 1 Cas9 guide cloning the following template was used:
  Illustrative target sequence with PAM underlined.
  TACGTGGACTAGTCAGTTAGNGG.
  *Ta*U6 promoter:
  Forward oligo (cloning overhang in italics).
  5’ *CTTG*TACGTGGACTAGTCAGTTAG 3’
  Reverse oligo (cloning overhang in italics).
  5’ *AAAC*CTAACTGACTAGTCCACGTA 3’
  *Ta*U3 and *Hv*U3 promoter:
  Forward oligo (cloning overhang in italics).
  5’ *AGCA*TACGTGGACTAGTCAGTTAG 3’
  Reverse oligo (cloning overhang in italics).
  5’ *AAAC*CTAACTGACTAGTCCACGTA 3’
  For level 1 Cas12a guide cloning the following template was used:
  Illustrative target sequence with PAM underlined.
  *TTTV*TCCATAGTGAGAAGAGGTGTGAG.
  V2 guide architecture:
  Forward oligo (cloning overhang in italics).
  5’ *AGAT*TCCATAGTGAGAAGAGGTGTGAG 3’
  Reverse oligo (cloning overhang in italics).
  5’ *GGCC*CTCACACCTCTTCTCACTATGGA 3’
  V3 guide architecture:
  Forward oligo (cloning overhang in italics).
  5’ *AGAT*TCCATAGTGAGAAGAGGTGTGAG 3’
  Reverse oligo (cloning overhang in italics).
  5’ *ATTA*CTCACACCTCTTCTCACTATGGA 3’
5. Prior to cloning oligos in level 1 accepters, oligo pairs were prepared at 2 μm in hybridisation buffer (10 mM Tris HCl pH 7.5, 50 mM NaCl, 1mM EDTA) and put into a heat block at 95 °C for 3 min before switching off and allowing to return to room temperature. 2 µl of the hybridised pair was added to 100ng of relevant guide accepter with 10 units of *BsaI*, 400 units T4 ligase, 0.1 mg/ml BSA, 1x T4 ligase buffer in a final volume of 10 µl. Cycling and transformation of *E.coli* was carried out as in 3 above.
6. 1 or 2 white colonies were picked at each stage of cloning, grown overnight in Luria broth liquid media before extracting plasmid using a Qiagen miniprep kit. Integrity was checked first by appropriate restriction digest followed by Sanger and/or whole plasmid sequencing.
7. Validated final level 2 plasmids were transformed into AGL1 *Agrobacterium* competent cells according to manufacturer instructions (Intact genomics) and again validated for integrity before being used in barley and wheat transformation.

### Generation of transgenic plants

1. Barley variety Golden Promise and wheat variety Fielder were transformed using previously reported protocols [12, 13].
2. In wheat experiments with Dexamethasone induction of GRF-GIF, dexamethasone was added to media from the point of *Agrobacterium* inoculation until shoot isolation at a final concentration of 10µM.

### Screening for mutations

1. Leaf sections of approximately 1cm^2^ from regenerated, rooted plants were used to extract genomic DNA using a Qiagen DNeasy kit and was used as template in PCR to amplify target loci. PCR was done using a Go Taq hot start polymerase kit (Promega) according to manufacturer instructions.
2. For Sanger sequencing, amplicons were cleaned first using ExoSAP-IT clean up (Thermo Fisher) according to manufacturer instructions. Sequencing was done according to commercial provider requirements. ABI sequence files were aligned to wild type reference sequence using the alignment tool in Benchling to identify the presence of mutagenesis. Sanger ABI files for lines identified as mutagenised are deposited at https://zenodo.org/doi/10.5281/zenodo.10731786.
3. Amplicons for Illumina sequencing had an additional round of PCR to add generic tails before being pooled in a commercial service providers library. Amplicon read pairs were mapped to the three loci of target genes, chr6Hg0653951, chr7Hg0684671 and chr2Hg0138701 using bwa [31] mem with default parameters. A custom script was used to process the resulting SAM files. Briefly, read pairs were filtered by both mates starting at boundaries defined by amplicon library preparation. Those starting points were determined from the mapping by finding the starting points of the majority of read pairs in each locus. Next, patterns of deletions in the loci were determined by parsing of CIGAR strings in SAM files and number of read pairs supporting each deletion-pattern were recorded. For overall report per sample, the percentage of usable reads supporting a wild-type allele was used as an output. Script was written in Java. Scripts are available at github.com/steuernb/Barley_Cas12a_AmpliconAnalysis. Illumina sequence data has been published via EBI PRJEB73387.

**Table 3:** Restriction enzymes required during the 4 different cloning steps in GoldenGate digestion-ligation reactions

Cloning step	Restriction enzymes in Golden Gate cloning reaction
Oligo pairs into level 1 guide accepters	*BsaI*
Level 1 to level 2	*BsaI* & *BpiI*
Level 1 to level M	*BpiI*
Level M to level 2	*BsaI* & *BpiI*

### Gene identifiers

1. Barley gene identifiers are in accordance the Toulouse INRA genome browser, a public Golden Promise genome database accessed at: https://bbric-pipelines.toulouse.inra.fr/myGenomeBrowser?browse=1&portalname=Hordeum_vulgare&owner=cyril.libourel@univ-tlse3.fr&key=OmReijye.
2. The *EnsemblPlants* gene identifiers for the Fielder wheat genes targeted are: TaGW2: TraesCS6A02G189300, TraesCS6B02G215300, TraesCS6D02G176900. TaGW7: TraesCS2A02G176000, TraesCS2B02G202300, TraesCS2D02G183400. RLK: TraesCS7A02G264400, TraesCS7B02G162500, TraesCS7D02G265400.

## Discussion

### Optimal Cas9 CDS

We systematically tested different *Sp*Cas9 and *Lb*Cas12a CDS’s and guide architectures in barley to define highly efficient Cas9 and Cas12a systems which we further tested in wheat where they were also highly effective. First, we compared *Hs*Cas9, *At*Cas9 + 1int & *Zm*Cas9 + 13int CDS’s and found the latter to be the top performer. *Hs*Cas9 and *Zm*Cas9 + 13int were previously compared in *Arabidopsis* and *N.benthamiana*, where the latter gave a much higher rate of editing. Western blots were performed in *N.benthamiana* by these authors who found that a much higher concentration of Cas9 protein accumulated in plant tissue when *Zm*Cas9 + 13int was used compared to *Hs*Cas9 [11]. Despite *At*Cas9 + 1int possessing different codon usage and some variation in nuclear localisation signals relative to *Hs*Cas9 it is likely intron mediated enhancement increased the abundance of overexpressed Cas9 in barley, resulting in more efficient editing (*At*Cas9 + 1int 88% >33% *Hs*Cas9). It is possible that the greater number of introns possessed by *Zm*Cas9 + 13int (13) compared to *At*Cas9 + 1int (1) explains the elevated performance of *Zm*Cas9 + 13int that we observed. It is notable that the dicotyledon derived introns inserted into these CDS’s are spliced effectively in the monocotyledon’s barley and wheat, indicating conservation of mechanism across these distant relatives. The benefit to enhancing transgene expression from the insertion of introns has been previously reported [32–34].

### Optimal Cas9 guide architecture

We compared three different Cas9 guide architectures in barley. The most frequently reported version (A) where individual guides in an array are driven by their own polymerase 3 promoter and followed by a short poly T terminator, performed the best. Here all three target genes were mutagenised in 100% of lines tested. This was a narrow improvement over architecture B where all three targets were mutagenised in 90% of lines. Architecture C, using CSY4 cleavage to separate multiple guides in a single polymerase 2 derived transcript, was the least effective here. 50% of the four T0 lines created were mutagenised in all three genes. Previously we have had good results with multiplex editing in *Medicago truncatula* [21] using a CSY4 cleavage system. In *Medicago*, the guide architecture was identical, however, the co-expressed CSY4 formed the 5’ terminal section of a fusion to an *Arabidopsis* codon optimised Cas9 CDS [19], the two being linked by a P2A skipping sequence. In barley architecture C, the CSY4 was co-expressed on the same transcript as the guide array and not the Cas9. It may be that this variation is less effective than the *Medicago* version. It was observed that construct C gave very few transgenic lines even after several repeat experiments which may result from some level of toxicity of the CSY4 protein to barley. This makes a fair comparison to architectures A and B impossible. We are aware of other anecdotal reports where CSY4 toxicity in plants is suspected.

Because the *Zm*Cas9 + 13int CDS/guide architecture A performed best in barley, we trialled it in the close relative wheat, where we targeted a single (*RLK*) gene present in all 3 subgenomes. The 4 guides used each targeted all 3 *RLK* copies. More than 90% of the 48 T0 lines tested were mutagenised in each of the 3 subgenomes (A-98%/B-94%/D-92%) confirming this Cas9 system as a great performer in wheat as well as barley.

### Optimal Cas12a CDS

For Cas12a we compared six CDS variants by targeting three barley genes (chr6Hg0653951, chr7Hg0684671 and chr2Hg0138701). Surprisingly we found no editing activity at all resulting from *Os*Cas12a. We found that the human codon optimised version (*Hs*Cas12a) worked better than the *Arabidopsis* codon optimised version (*At*Cas12a) having overall mean efficiencies of 44% and 27% respectively, although neither resulted in mutagenesis of chr7Hg0684671. When the “temperature tolerant” mutation D156R was added to both of these CDS’s then their performances were improved as expected giving mean efficiencies of tt*Hs*Cas12a: 72% & tt*At*Cas12a: 53%. This boost in performance by adding D156R enabled the mutagenesis of the recalcitrant target gene chr7Hg0684671 to 33% and 17% with tt*Hs*Cas12a and tt*At*Cas12a respectively. The most effective Cas12a CDS was when the tt*At*Cas12a had 8 short *Arabidopsis* introns inserted into it in tt*At*Cas12 + 8int. Overall mean efficiency rose to 87% and even the recalcitrant target chr7Hg0684671 had 68% mutations. It is surprising that *Os*Cas12a seems to lack functionality in barley as it has been used previously with success in *Zea mays*, another monocotyledon, although efficiency cannot really be inferred from the three T0 plants reported on [35]. However, when *Os*Cas12a was used in wheat, efficiency appeared rather low with only two of fifty-one T0 plants created containing mutations [29]. This was at the target site of GW7T14, the same guide which we used later here in conjunction with tt*At*Cas12a + 8int/V3 guide architecture to achieve 86% T0 editing. To make the original *Os*Cas12a CDS [22] compatible with our cloning system it was necessary to make 4 silent nucleotide changes. It is possible that despite selecting frequently used codons as these synonymous substitutions we inadvertently affected the functionality of the *Os*Cas12a CDS negatively, potentially accounting for the lack of function seen in barley. As in barley, we found that tt*At*Cas12a + 8int performed better than tt*Hs*Cas12a in wheat, giving an average percentage (across *Ta*GW2 & *Ta*GW7 A, B,D copies) of 72% compared to 46% mutagenised T0 plants.

It is likely that insertion of introns into the tt*Hs*Cas12a CDS would boost efficiency beyond that obtained with tt*At*Cas12a + 8int as tt*Hs*Cas12a outperformed tt*At*Cas12a in our experiments.

### Dissecting the role of introns in Cas12a CDS

We were interested in dissecting the roles of the 8 introns inserted into tt*At*Cas12a + 8int by making comparable constructs retaining one or more of the original eight. The results showed that the first intron did not substantially enhance mutagenesis resulting in near identical overall efficiency to the no intron version (tt*At*Cas12a: 53%, tt*At*Cas12a + 1int: 54%). The first 3 introns (tt*At*Cas12a + S1int) and the last two introns (ttAtCas12a + S3int) gave a very similar level of enhancement boosting the mean overall efficiencies to 67% and 66% respectively. The second 3 introns in tt*At*Cas12a + S2int gave the greatest enhancement effect in this dissection experiment, resulting in an overall mean efficiency of 84%. This was very close to the overall mean efficiency when all 8 introns were present in tt*At*Cas12a + 8int (87%). However, when the recalcitrant target chr7Hg0684671 was observed it was apparent that all eight introns in tt*At*Cas12a + 8int are more effective than just the second 3 introns in tt*At*Cas12a + S2int (mean efficiencies of 68% & 59% respectively). It is reported elsewhere that differences in editing efficiencies between Cas12a components are most apparent where the target is typically edited to a low level with the least efficient variant under test [4]. Accordingly, it is not surprising that the difference in efficiency between tt*At*Cas12a + 8int & tt*At*Cas12a + S2int is most visible at chr7Hg0684671. It may be that some of the differences in efficiency we observed between intron variants are because some introns have more of an enhancing effect than others, a feature which has been reported before in relation to intron mediated enhancement (IME) [36]. These authors developed an algorithm based on motifs identified in introns known to elevate gene expression. The output score is more positive the greater the predicted IME effect. Running our intron sequences through this publicly available program gave a negative score for the first intron and a positive score for the second and third intron. This may explain the lack of IME we saw in tt*At*Cas12a + 1int (1%) which contained only the first intron, compared to tt*At*Cas12a + S1int, which contained the first, second and third introns and gave an overall enhancement of 14%. However, in tt*At*Cas12a + S2int all three introns have a negative score which does not correspond to the high degree of enhancement observed (31%).

One mechanism proposed to explain IME is based on components of the spliceosome preventing post transcriptional gene silencing (PTGS) [32]. It was shown that intron splicing reduced the abundance of siRNAs by making transcripts less effective substrates for RNA-dependent RNA polymerase 6 (RDR6). This may explain why the full 8 introns present in tt*At*Cas12a + 8int had the greatest enhancement in mutagenesis, based on having the most introns and splicing occurring, therefore greater potential for RDR6 inhibition and prevention of PTGS. Another mechanism could involve a synergistic interplay between introns, leading to increased splicing efficiency, and in turn to more abundant Cas12a mature mRNA. Ultimately IME is highly likely to result in a greater abundance of the Cas12a protein. Previously more Cas9 protein was seen when 13 introns were inserted into the ZCas9 CDS than in the intron free version [11]. Our results show that there is rationale to adding multiple introns to Cas nuclease CDSs. None of the intron combinations tested had a negative effect on efficiency and the benefits of IME were cumulative and greatest in the full eight intron Cas12a version. As there is no reliable way to predict the best intron and CDS insertion site to maximise IME that we are aware of, multiple introns can be used to circumvent this. This is likely to be a useful design parameter for transgenes other than Cas nucleases and to be of interest to anyone trying to maximise expression of their favourite gene in plants.

### Combinatorial effect of D156R and introns in Cas12a CDS

We were also interested to see if the combined effect of D156R and 8 introns in tt*At*Cas12a + 8int was greater than the sum of the singular parts in tt*At*Cas12a and *At*Cas12a + 8int. It was only possible to determine the gain in enhanced mutagenesis from the combined D156R/intron effect at the recalcitrant target chr7Hg0684671 because the singular D156R/intron effects were already so high in the other two targets, taking the summed efficiencies above 100%. The gain in efficiency at chr7Hg0684671 from the combinatorial effect was 48%. The combinatorial effect is likely to be multiplicative i.e. more than the sum of the individual parts if the D156R mode of action is at the protein structure level and the IME at the level of protein abundance. This is highly likely as D156R represents a change in the peptide sequence and the eight introns are likely to be increasing the abundance of the Cas12a protein, just as they were previously reported to do so for Cas9 [11], perhaps via inhibition of PTGS.

### Optimal Cas12a guide architecture

Recently, we were informed by a report in wheat which compared V2 & V3 Cas12a guide architecture in a protoplast system [28]. Like these authors, we found V3 performed better than V2 when we made the comparison in stably transformed barley & wheat lines. In barley the overall mean mutagenesis efficiency of V2 and V3 were similar at 87% and 90% respectively. However, considering only the most recalcitrant target gene chr7Hg0684671 the respective values were 68% and 90%, showing a clear benefit of V3 over V2. In wheat the average percentage of mutagenised T0 plants (across *Ta*GW2 & *Ta*GW7 A, B,D copies) rose from 72% with V2 to 90% with V3. Once again, this difference was most evident when just considering the most recalcitrant target, which in this case was *TaGW2*. Here the mean efficiency rose from 63% with V2 to 93% with V3.

### Multiplex mutagenesis with co-expressed Cas9 and Cas12a in barley

We were able to introduce both *Zm*Cas9 + 13int and tt*At*Cas12a + 8int on a single T-DNA to bring about effective co-nuclease mutagenesis in barley. We were successful in all attempts to simultaneously target two, three and four genes in multiplex experiments where four guides per gene were used. Between 32% and 100% of T0 lines were successfully mutagenised in all target genes for the four constructs tested. These efficiencies did not appear to consistently relate to the number of genes being targeted. In one comparison a construct targeting two genes was more efficient than a construct targeting four genes - construct 2 targeting two genes mutagenised all targets in 100% of T0 lines and construct 4 targeting four genes mutated 68% of T0 lines in all targets. In another comparison the reverse was true - construct 4 targeting 4 genes was more efficient (68%) than construct 1 (45%) targeting two genes. These variations in efficiencies are likely to reflect inherent differences in target accessibility. With up to sixteen guide cassettes plus two intronised Cas nuclease cassettes, T-DNA inserts were rather large, but despite their size transformed barley sufficiently well to give between 11 and 19 independent T0 lines per construct. Although a direct comparison between Cas9 and Cas12a mutagenic efficiency is not possible here due to the co-existing nucleases targeting different genes and/or loci within genes, both nucleases appeared to work equally well. For example, in lines containing construct 1 targeting chr5Hg045473 with x4 Cas12a guides and chr4Hg0361381 with x4 Cas9 guides, both genes were mutagenised in 45% of T0 plants. Similarly in lines containing construct 2 targeting chr6Hg0674791 with x4 Cas9 guides and chr6Hg0674101 with x4 Cas12a guides, 100% of T0 lines were mutagenised in both genes. The factor limiting the number of T0 lines mutated in all target genes was always the target gene mutagenised at the lowest rate for any particular construct, such that the more efficiently targeted genes were always edited in the lines where the less efficiently edited genes were mutagenised. This is likely to have implications in the chance outcomes of different mutant combinations. For example, it may be relatively simple to obtain lines where two of the most efficiently targeted of three genes are edited, but obtaining lines where just the most and least efficiently targeted genes are hit is likely to be more troublesome. If such mutant combinations are required, then it is likely to be necessary to use separate constructs targeting specific gene combinations. Co-expression of Cas9 and Cas12a will extend the number of editing options available, for example the ability to simultaneously target GC and AT rich regions could enable both promoter and exon mutagenesis to be done in the same plant. Cas9 is often used for frame shift loss of function resulting from small indels, whilst Cas12a is well suited to larger deletions appropriate to promoter elements. Using co-expression makes the two tasks achievable in one plant in a single generation. Recently co-expression of a Cas9 based cytosine base editor and Cas12a was used in tomato to give editing events which were free of the T-DNA used to deliver reagents [37]. Cas9 base editing allowed selection on herbicide containing media and regenerated plants contained Cas12a derived mutations at a second locus whilst not retaining the T-DNA for integration. The tools developed here could be taken further to facilitate such a system in wheat and barley, potentially allowing the production of transgene free first-generation edited plants.

Our preliminary experiments with GR-GRF-GIF indicate that this fusion gives Dex inducible benefits in terms of transformation enhancement in wheat. This option in our toolkit could be applied in varieties susceptible to GRF-GIF pleiotropic effects where undesirable phenotypes such as sterility are a problem. Anecdotal reports suggest this is more common in tetraploid varieties.

### Electronic supplementary material

Below is the link to the electronic supplementary material.

Additional file 1: Schematic of 15 constructs used in barley Cas9 coding sequence comparison.

Additional file 2: Tabular efficiency data for barley Cas12a coding sequence comparison.

Additional file 3: Schematic of three Cas9 guide architectures compared in barley.

Additional file 4: Tabular efficiency data for three Cas9 guide architectures in barley.

Additional file 5: Schematic of construct used to validate optimised Cas9 parts in wheat.

Additional file 6: Tabular efficiency data for optimised Cas9 parts in wheat.

Additional file 7: Schematic of constructs used to compare Cas12a coding sequences in barley.

Additional file 8: Tabular efficiency data for Cas12a coding sequences in barley.

Additional file 9: Cas12a coding sequence efficiency data in barley plotted individually for three target genes.

Additional file 10: Dissecting the role of introns in enhancing Cas12a mutagenesis efficiency. Schematic of constructs and plotted efficiency data.

Additional file 11: Legend for additional file 10.

Additional file 12: Tabular efficiency data for Cas12a intron dissection experiment.

Additional file 13: Plotted efficiency data for comparison of sum vs. combined effect of D156R and introns in enhancing Cas12a efficiency in barley.

Additional file 14: Tabular efficiency data of Cas12a sum vs. combined effect of D156R and introns in enhancing Cas12a efficiency in barley.

Additional file 15: Schematic of constructs used to compare V2 and V3 Cas12a guide architectures in barley.

Additional file 16: Tabular efficiency data of Cas12a V2 vs. V3 guide architecture in barley.

Additional file 17: Schematic of constructs used to validate optimised Cas12a parts in wheat.

Additional file 18: Tabular efficiency data validating optimised Cas12a parts in wheat.

Additional file 19: Schematic of constructs used in Cas9 and Cas12a co-mutagenesis in barley.

Additional file 20: Tabular wheat transformation efficiencies using dexamethasone inducible GRF-GIF module.

Additional file 21: Spreadsheet itemising toolkit components separated into level 1, level M and level 2 Golden Gate parts. Vector choices itemised according to the number of guides required in the final construct.

Additional file 22: Spreadsheet itemising the constructs guides and primers used in this study. Constructs numbered according to subsequent additional .gb file maps.

Additional files 23–62: Constructs used in this study, itemised in additional file 21.

## Data Availability

The raw datasets supporting the conclusions of this study are publicly available : Sanger ABI files are deposited at https://zenodo.org/records/10731787. Illumina sequence data has been published via EBI PRJEB73387. The processed datasets supporting the conclusions of this article are included in the article and its additional files.
