# Supplementary figures and images for: An optimised CRISPR Cas9 and Cas12a mutagenesis toolkit for Barley and Wheat
Source: Plant Methods. 2024 Aug 13;20:123. doi: 10.1186/s13007-024-01234-y (PMC11321142; doi:10.1186/s13007-024-01234-y)

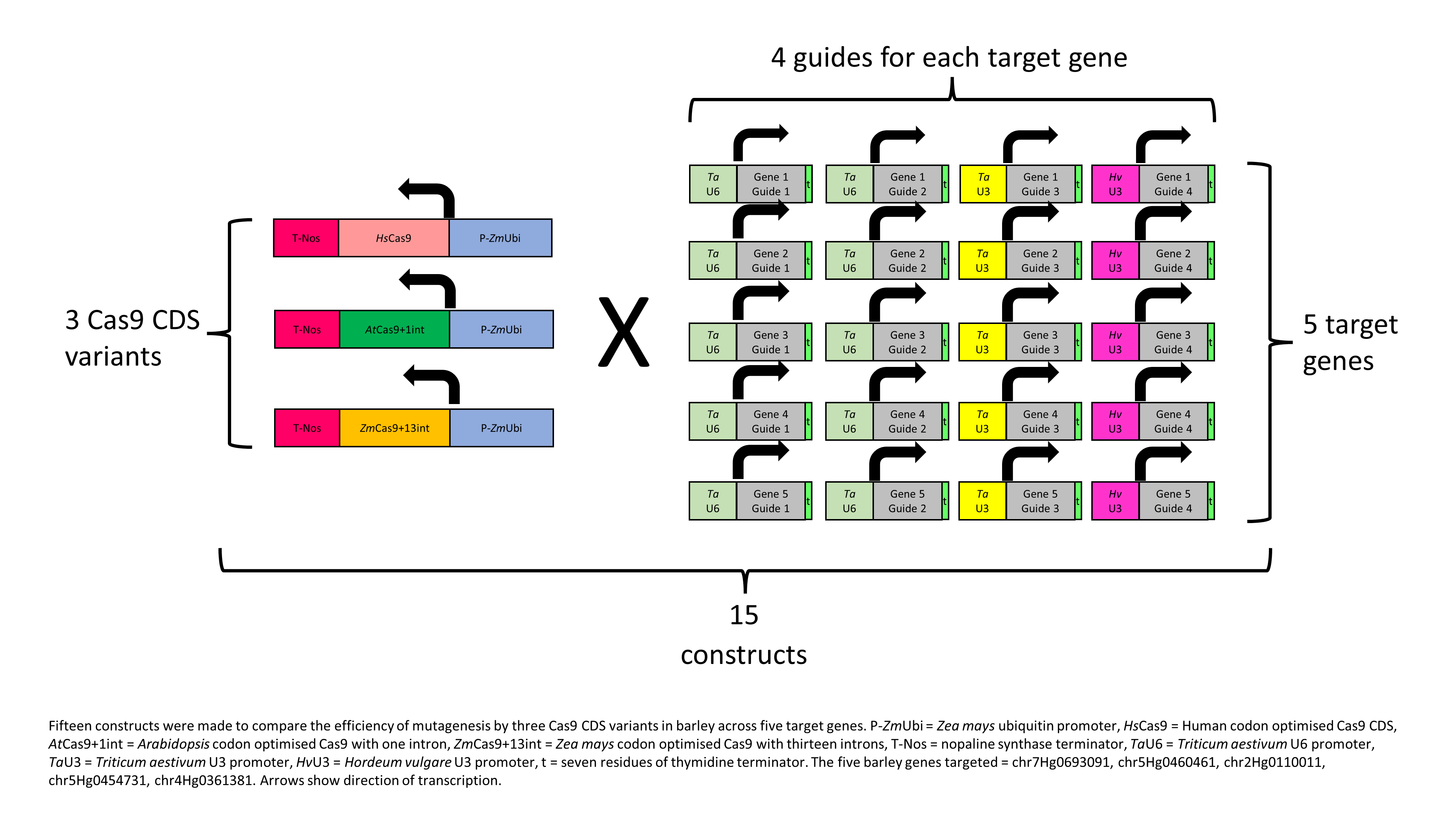

Supplement: Supplementary file 1 — Additional file 1: Schematic of 15 constructs used in barley Cas9 coding sequence comparison. [file 13007_2024_1234_MOESM1_ESM.tif]

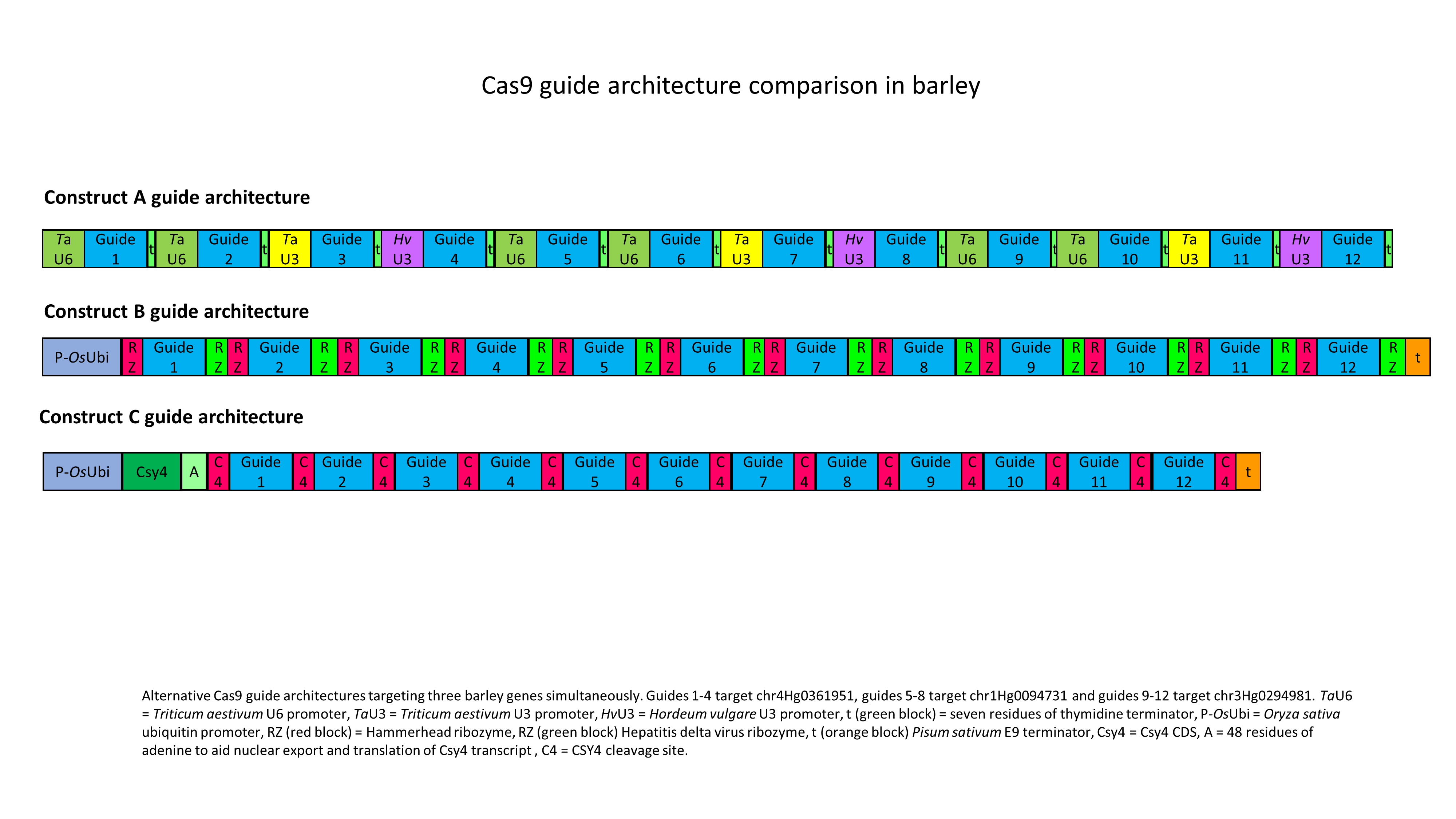

Supplement: Supplementary file 3 — Additional file 3: Schematic of three Cas9 guide architectures compared in barley. [file 13007_2024_1234_MOESM3_ESM.tif]

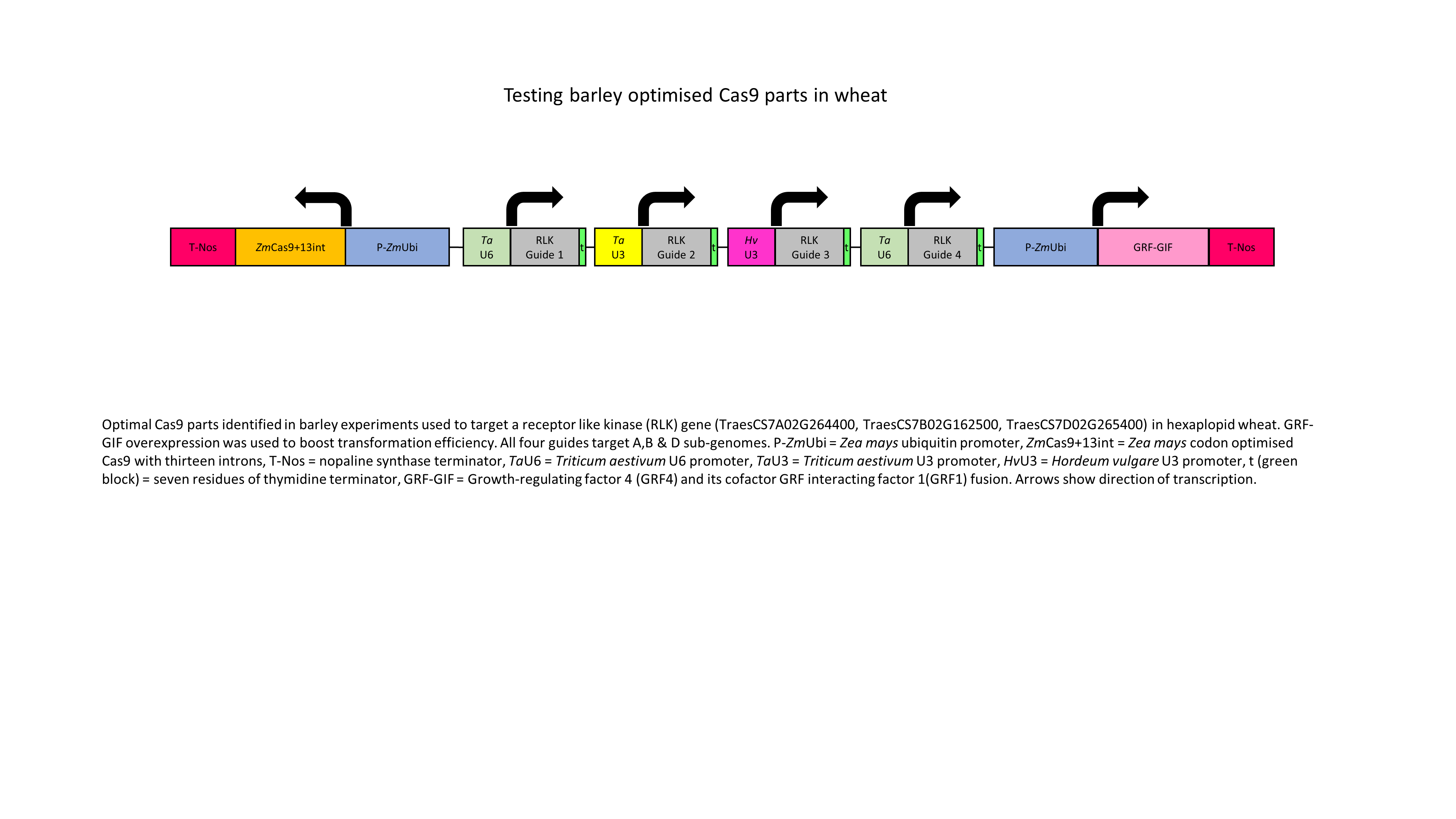

Supplement: Supplementary file 5 — Additional file 5: Schematic of construct used to validate optimised Cas9 parts in wheat. [file 13007_2024_1234_MOESM5_ESM.tif]

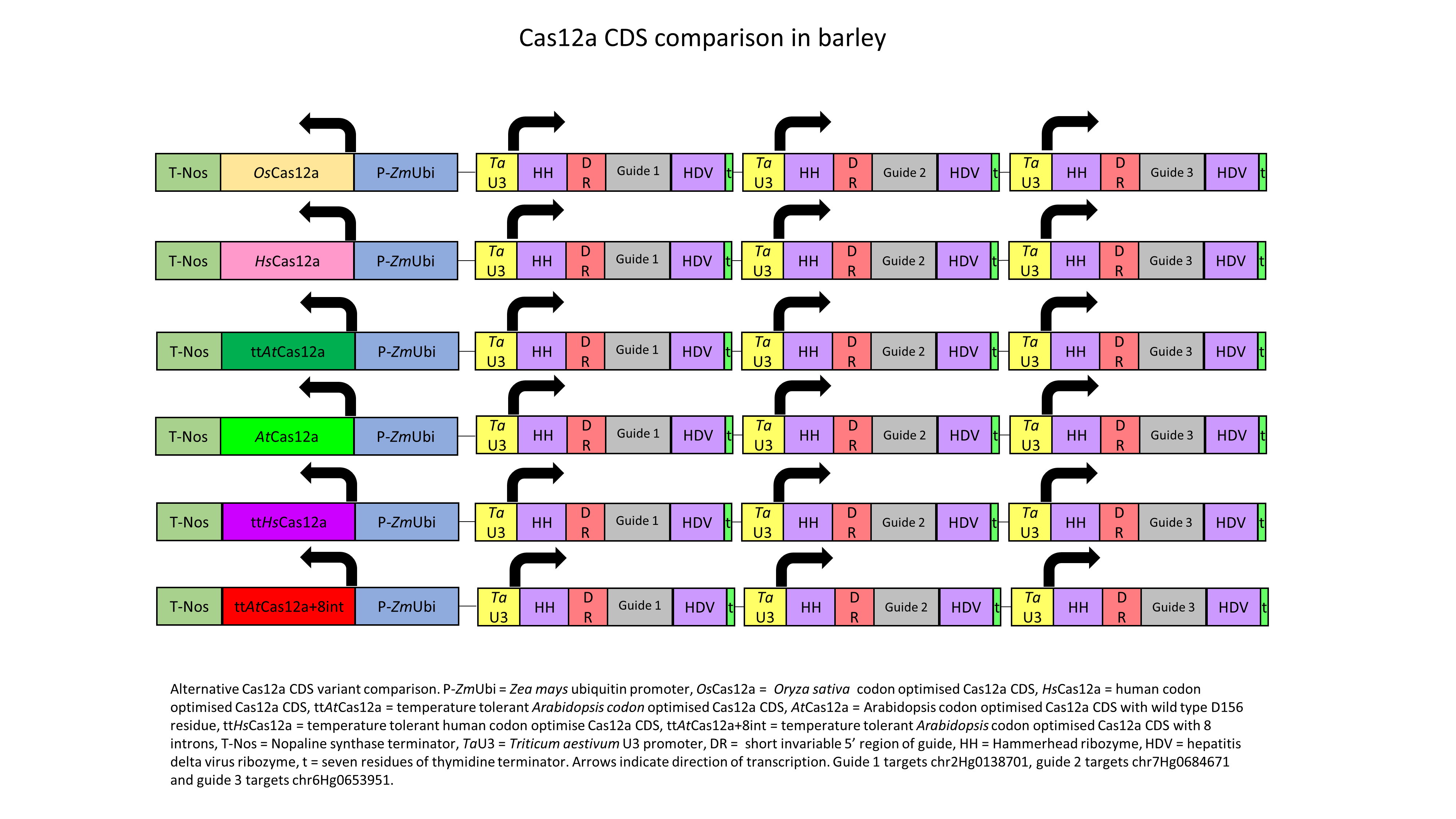

Supplement: Supplementary file 7 — Additional file 7: Schematic of constructs used to compare Cas12a coding sequences in barley. [file 13007_2024_1234_MOESM7_ESM.tif]

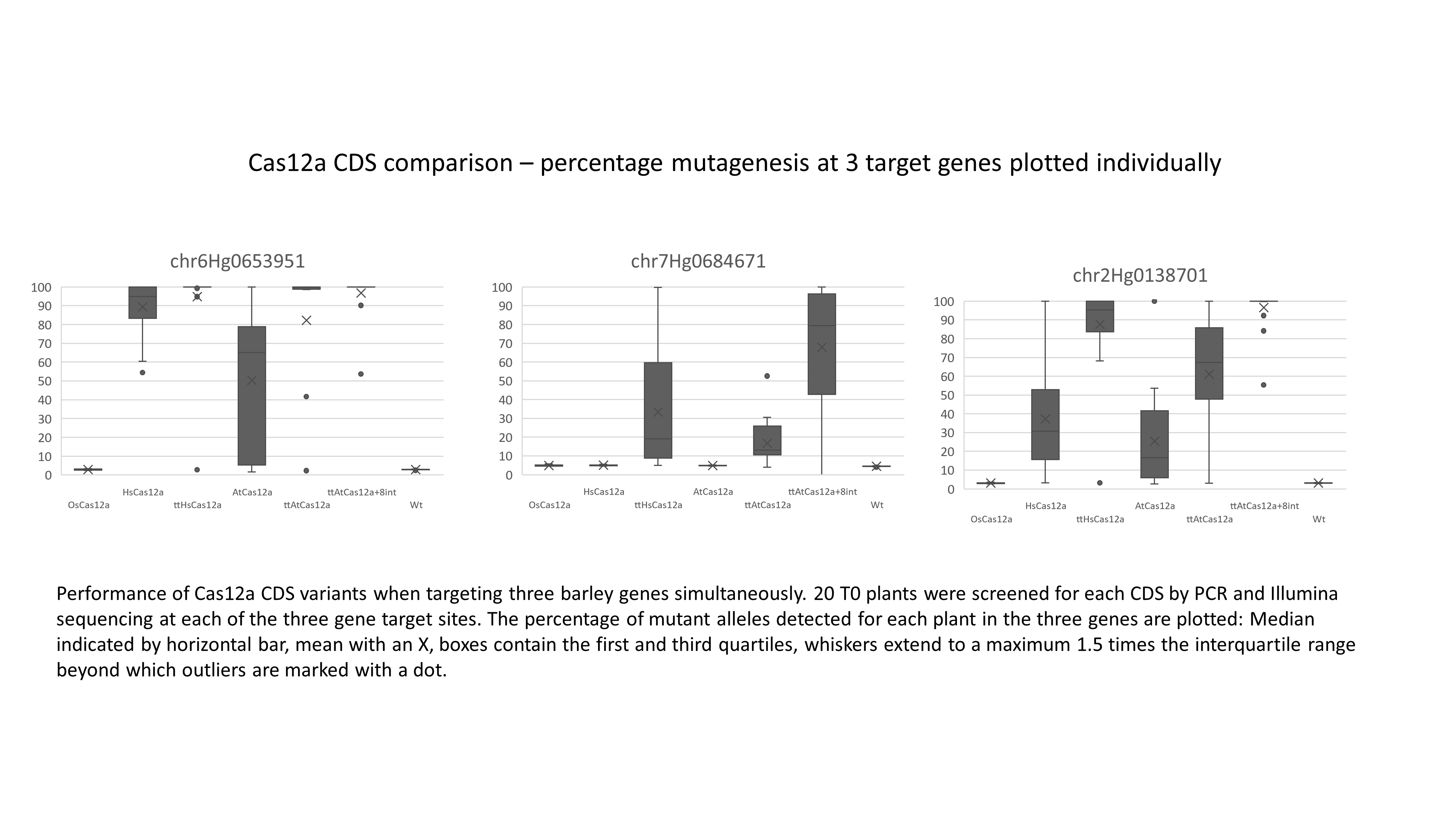

Supplement: Supplementary file 9 — Additional file 9: Cas12a coding sequence efficiency data in barley plotted individually for three target genes. [file 13007_2024_1234_MOESM9_ESM.tif]

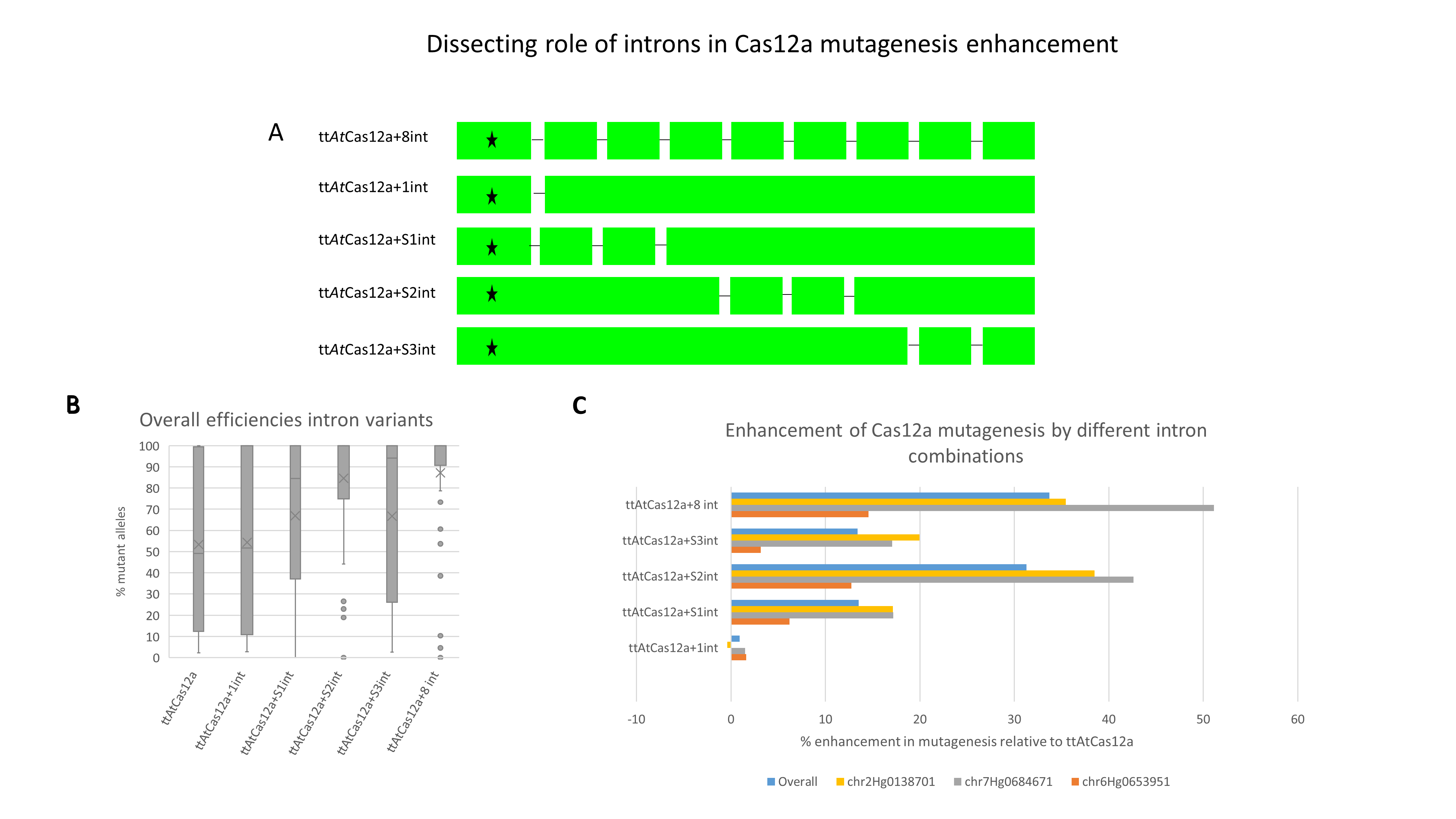

Supplement: Supplementary file 10 — Additional file 10: Dissecting the role of introns in enhancing Cas12a mutagenesis efficiency. Schematic of constructs and plotted efficiency data. [file 13007_2024_1234_MOESM10_ESM.tif]

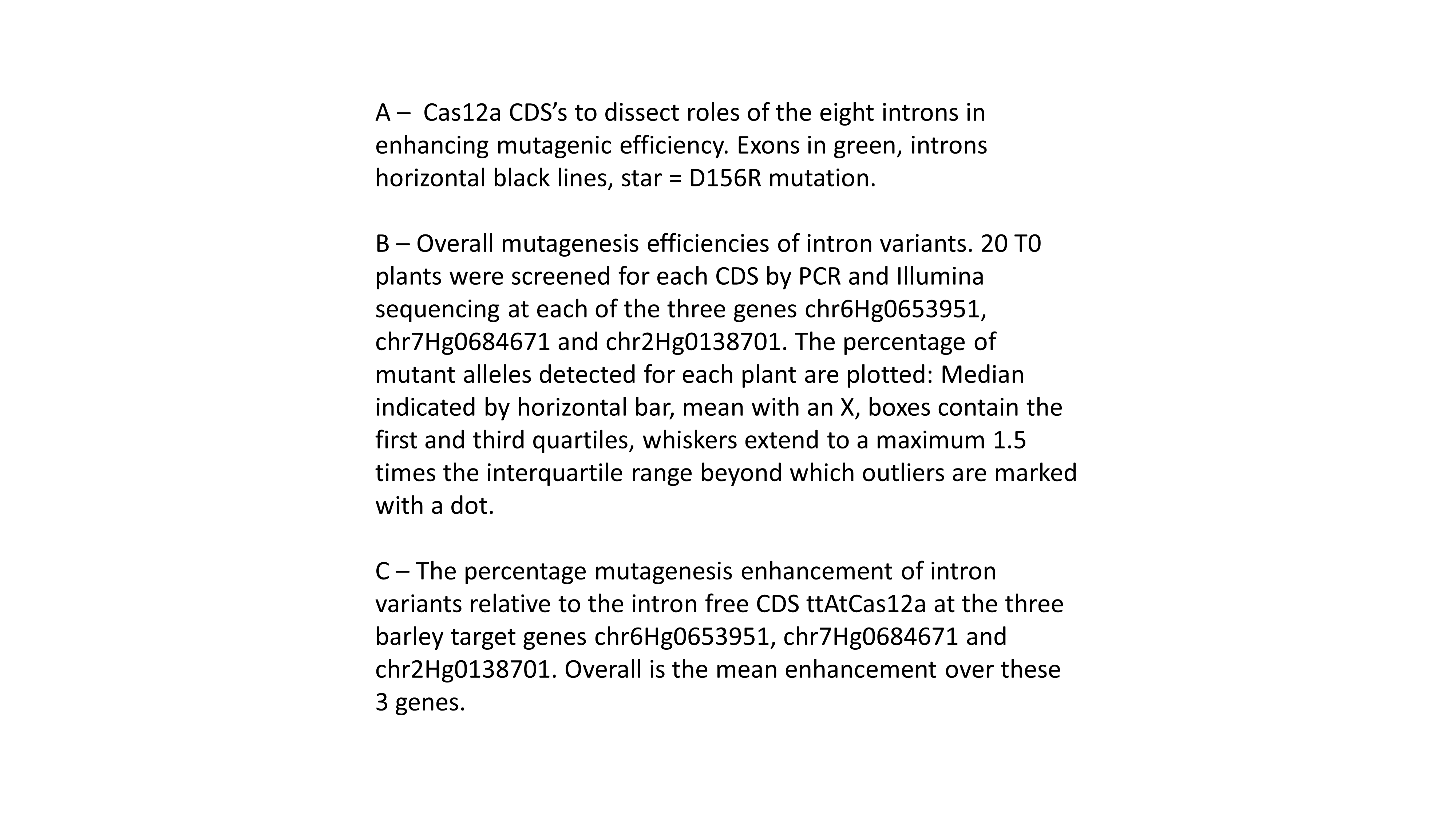

Supplement: Supplementary file 11 — Additional file 11: Legend for additional file 10. [file 13007_2024_1234_MOESM11_ESM.tif]

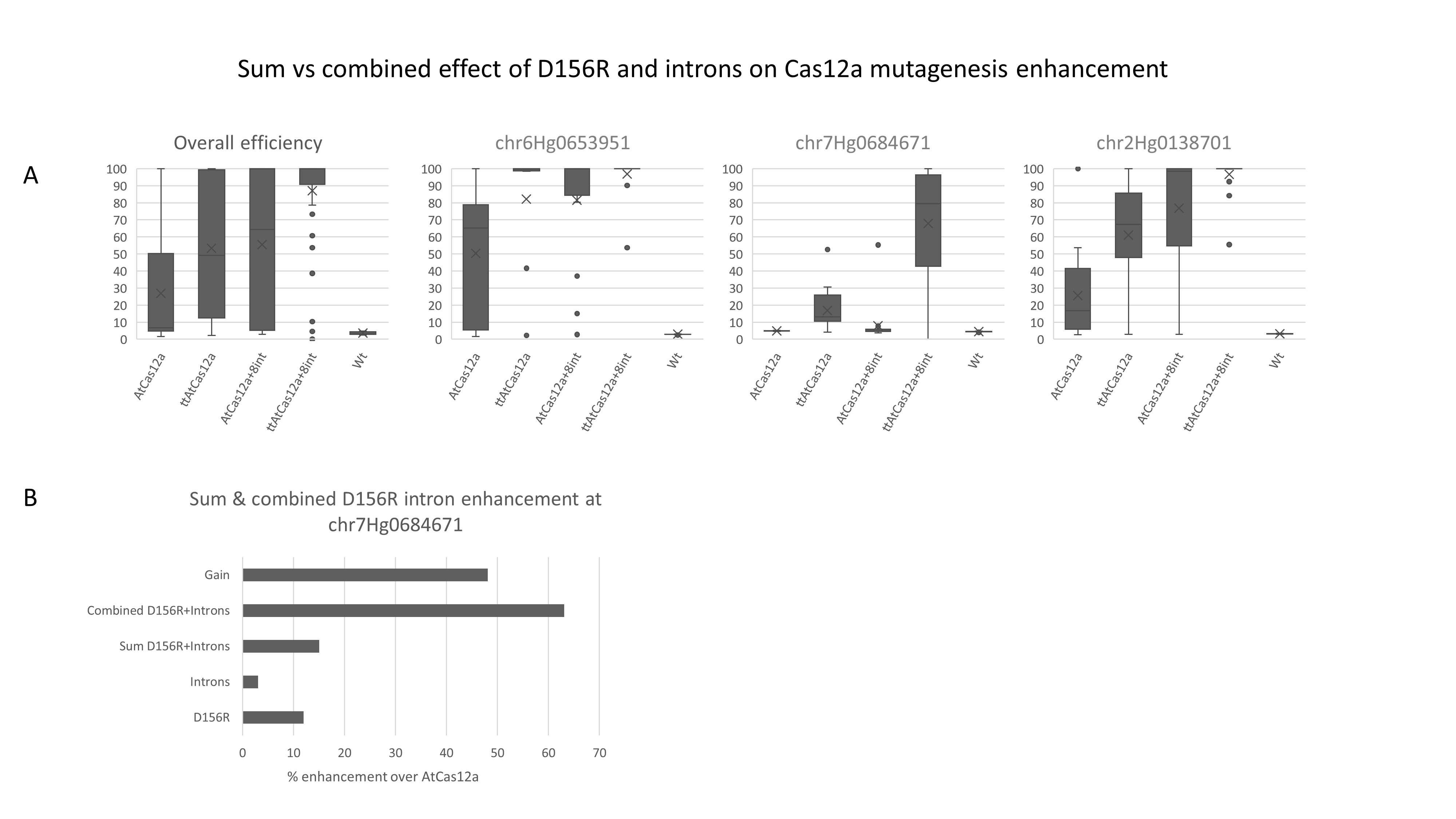

Supplement: Supplementary file 13 — Additional file 13: Plotted efficiency data for comparison of sum vs. combined effect of D156R and introns in enhancing Cas12a efficiency in barley. [file 13007_2024_1234_MOESM13_ESM.tif]

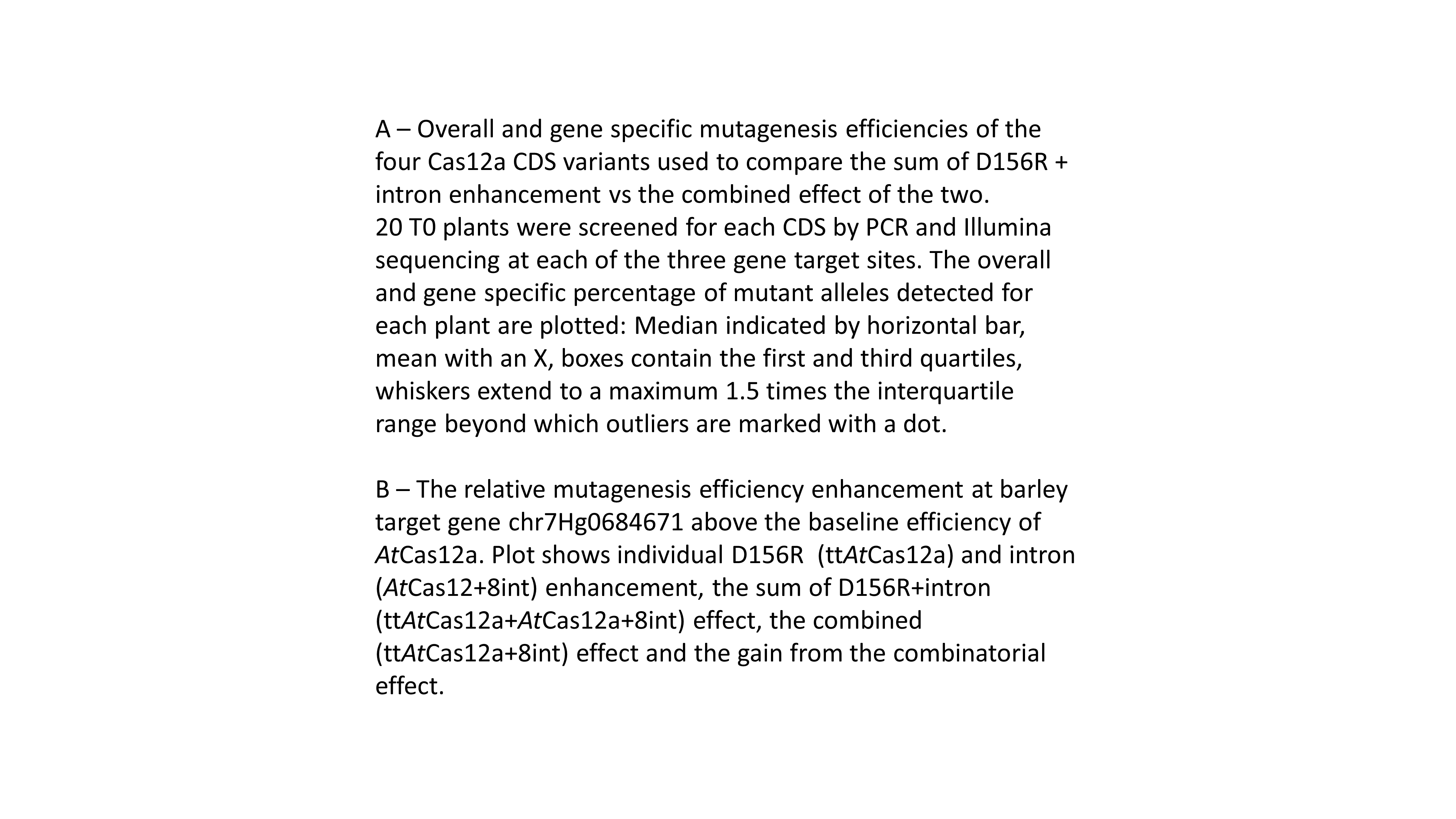

Supplement: Supplementary file 14 — Additional file 14: Tabular efficiency data of Cas12a sum vs. combined effect of D156R and introns in enhancing Cas12a efficiency in barley. [file 13007_2024_1234_MOESM14_ESM.tif]

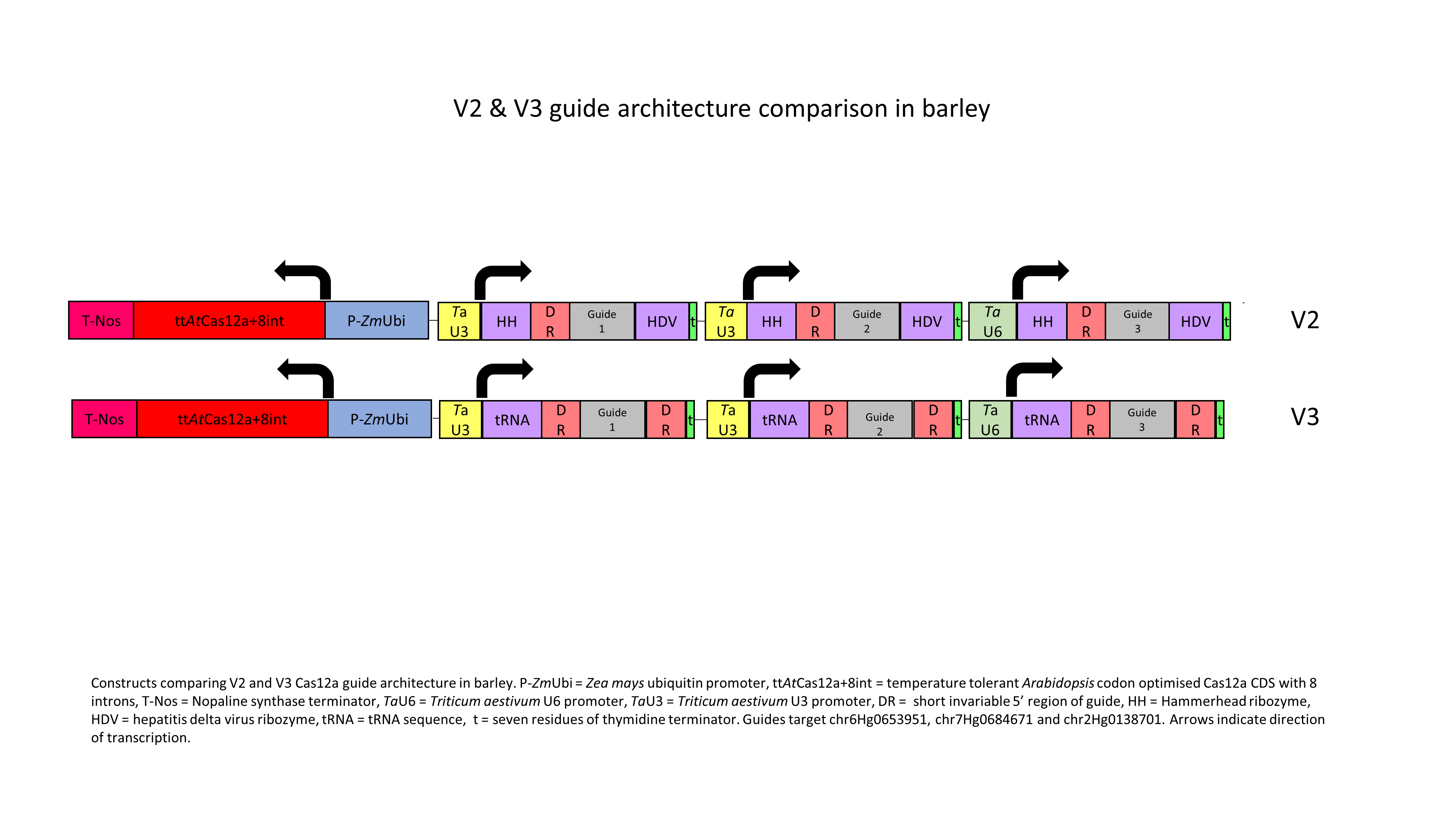

Supplement: Supplementary file 16 — Additional file 16: Tabular efficiency data of Cas12a V2 vs. V3 guide architecture in barley. [file 13007_2024_1234_MOESM16_ESM.tif]

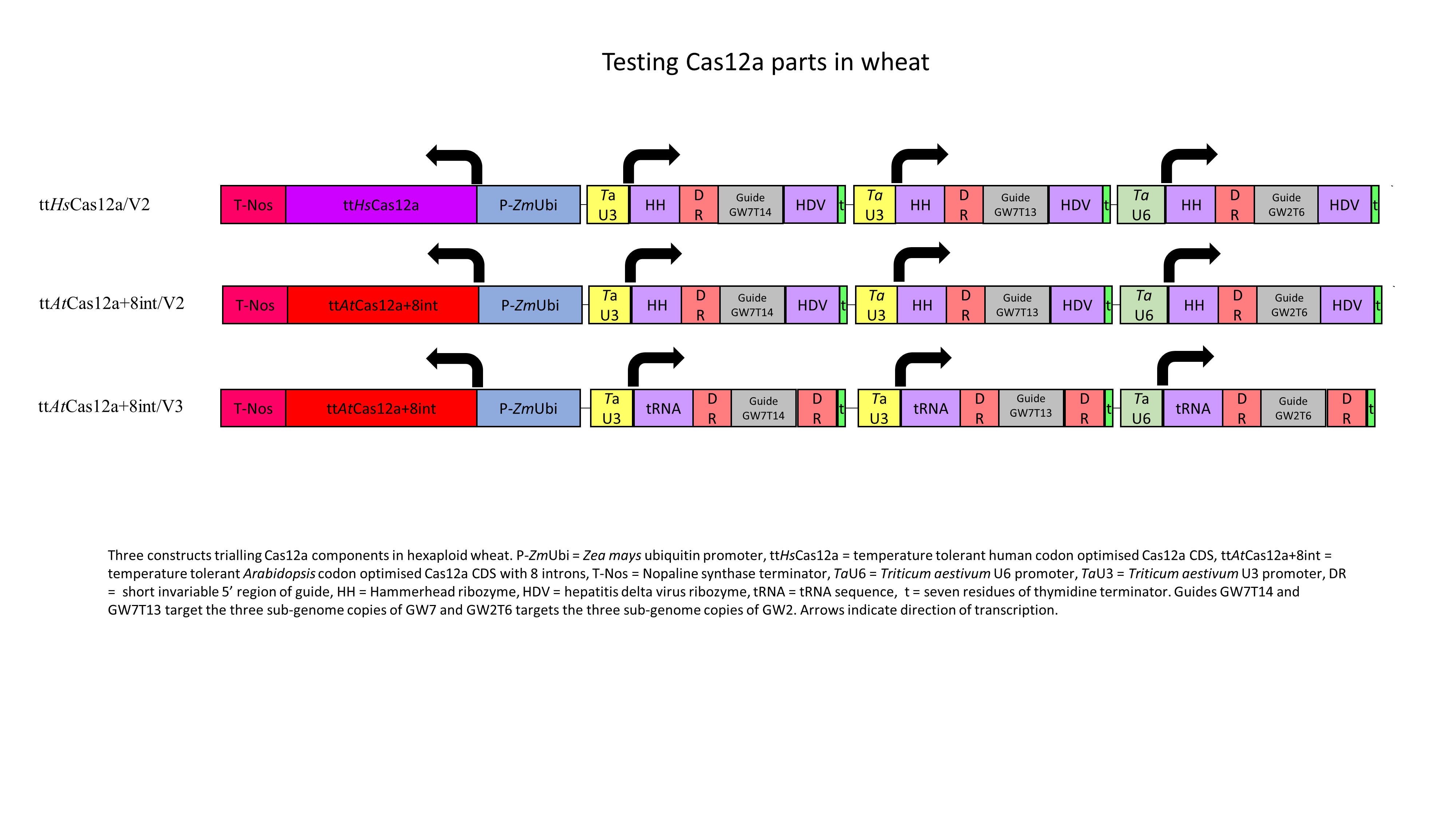

Supplement: Supplementary file 18 — Additional file 18: Tabular efficiency data validating optimised Cas12a parts in wheat. [file 13007_2024_1234_MOESM18_ESM.tif]

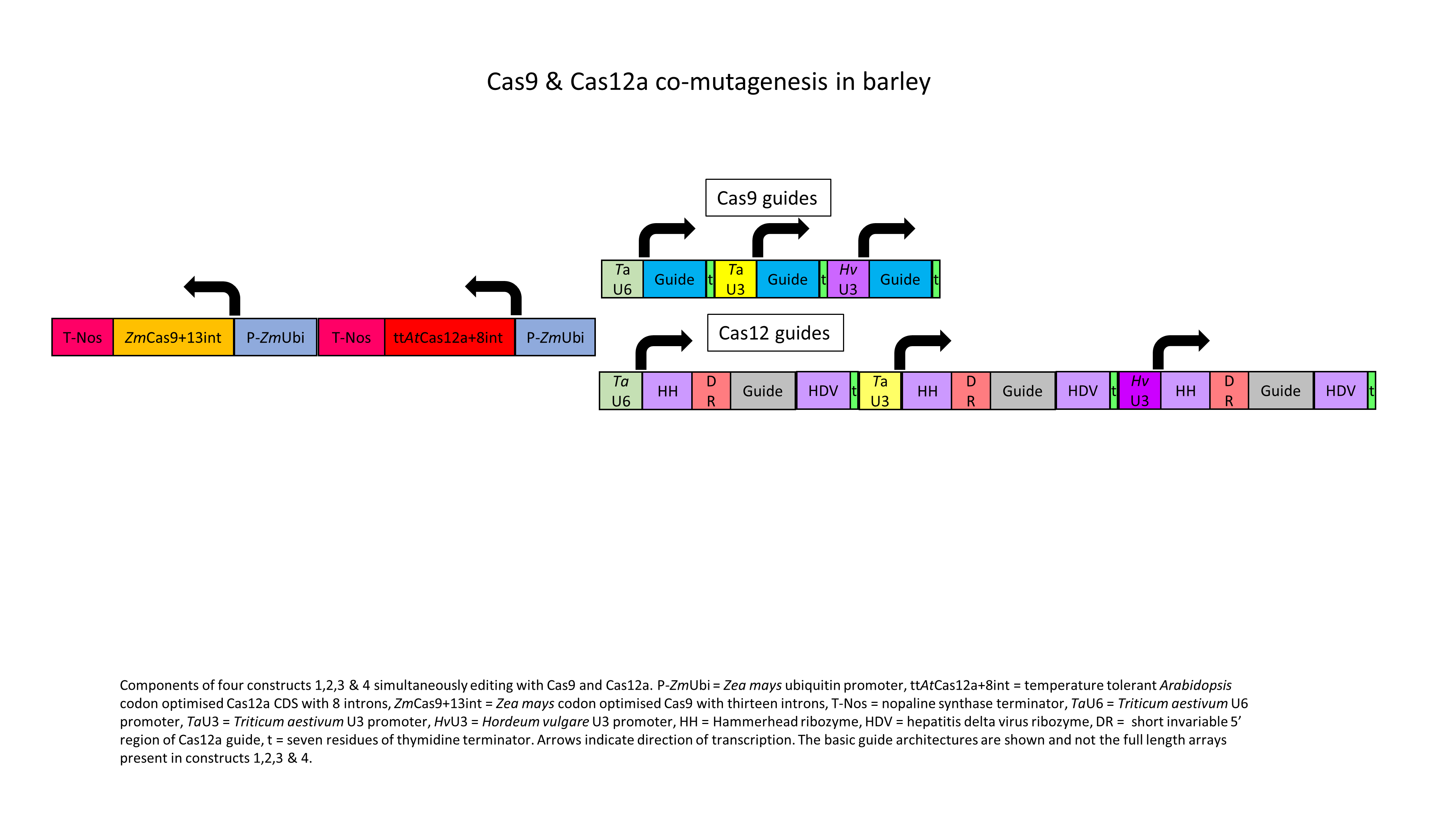

Supplement: Supplementary file 20 — Additional file 20: Tabular wheat transformation efficiencies using dexamethasone inducible GRF-GIF module. [file 13007_2024_1234_MOESM20_ESM.tif]
